# Mesostructured Nonwovens with Supramolecular Tricycloquinazoline Nanofibers as Heterogenous Photocatalyst

**DOI:** 10.1002/smsc.202300160

**Published:** 2023-12-10

**Authors:** Dennis Schröder, Christian Neuber, Ulrich Mansfeld, Klaus Kreger, Hans-Werner Schmidt

**Affiliations:** ^1^ Macromolecular Chemistry I and Bavarian Polymer Institute University of Bayreuth 95440 Bayreuth Germany

**Keywords:** mesostructured nonwovens, photocatalysis, physical vapor deposition, supramolecular nanofibers, tricycloquinazoline

## Abstract

Functional supramolecular nanostructures are a promising class of materials, which can be used as potential heterogeneous photocatalysts in water. Self‐assembly to nanoobjects in solution typically requires large solubilizing groups linked to the photoactive building block, and possibly hampers access to the photocatalytic active sites. Herein, a straightforward method to fabricate supramolecular nanofibers based on the disclike tricycloquinazoline (TCQ) by physical vapor deposition (PVD) is reported. It is demonstrated that TCQ can be assembled on different substrates into supramolecular nanofibers with diameters of about 70 nm resulting in densely packed fiber layers. With optimized conditions, the evaporation time allows full control over the fiber length and the absorbance of the TCQ fiber layer. A bottlebrush‐like morphology with TCQ nanofibers is realized using glass‐microfiber nonwovens as porous support. These mesostructured nonwovens can be used as photocatalysts for the degradation of rhodamine B in a batch process in water where the morphology remains intact after the reaction. After photocatalytic degradation of rhodamine B or tetracycline under continuous flow conditions, the supramolecular TCQ nanofibers still remain on the support. These findings demonstrate that PVD is a feasible approach to achieve functional mesostructured nonwovens with controlled morphology for use and reuse in catalytic applications.

## Graphical abstract

Physical vapor deposition is a straightforward method to fabricate well‐defined supramolecular nanofibers with full control over the fiber length. Using tricycloquinazoline as a molecular building block and glass‐microfiber nonwovens as a porous support, mesostructured nonwovens with a bottlebrush‐like morphology are realized, which can be used as reusable photocatalyst for the degradation of pollutants such as rhodamine B and tetracycline.

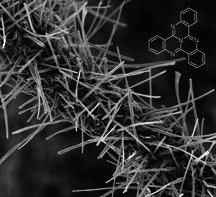

## 1 Introduction

Functional supramolecular architectures via self‐assembly of defined molecular building blocks have attracted significant attention in the field of materials science, because of their tailored and unique property profile.^[^ ^1^ ^]^ As a result, the development of functional supramolecular materials holds great potential in various fields, including biomedical applications,^[^ ^2^ ^]^ drug delivery,^[^ ^3^ ^]^ photovoltaics,^[^ ^4^ ^]^ energy storage,^[^ ^5^ ^]^ and (photo)catalysis.^[^ ^6^, ^7^ ^]^ In particular, photocatalysis has emerged as a rapidly evolving research area for solar fuels,^[^ ^6^, ^7^, ^8^, ^9^, ^10^ ^]^ chemical transformations,^[^ ^11^ ^]^ and environmental remediation.^[^ ^8^, ^12^, ^13^ ^]^ In this context, functional supramolecular materials offer intriguing prospects for enhancing the performance of photocatalytic systems due to their defined morphology on the nanoscale. The potential of photocatalytic supramolecular materials has already been demonstrated for various systems based on chromophoric cores such as perylene bisimides,^[^ ^12^, ^13^, ^14^ ^]^ porphyrins,^[^ ^10^, ^15^ ^]^ quinacridones,^[^ ^16^ ^]^ ullazines,^[^ ^17^ ^]^ and heptazines.^[^ ^9^ ^]^ Through precise control of non‐covalent interactions, these materials can exhibit a high surface‐to‐volume ratio, tunable light absorption, efficient charge separation, and enhanced catalytic activity.

Self‐assembly of molecular building blocks via directed secondary interactions into supramolecular nanoobjects, in particular nanofibers, requires typically a solution‐based process. Several parameters are decisive for the preparation of specific aggregates with defined morphology including the molecular structure, solvent, concentration, and processing window. The interplay between these parameters to realize defined nanoobjects often demands large solubilizing groups linked to the periphery of the molecular building blocks. These photo‐inactive solubilizing groups may hamper access to the π‐conjugated core, which is necessary to form the photocatalytic active species.

Apart from these considerations, self‐assembled objects on the nanoscale after the photocatalytic process in solution cannot be easily removed, redispersed, and reused, which is due to their small size and their potential to form larger agglomerates during the photocatalytic process and the removal step. Unsupported nanofibers may also hamper their use in continuous flow processes, because the release of the nanoobjects into the environment is difficult to control. In contrast, fixated supramolecular nanofibers on a macroscopic support are beneficial because the nanoscopic heterogeneous photocatalyst can be conveniently removed, reused, and recycled in this way, while its morphology is maintained. Moreover, the use of porous supports allows to apply continuous flow processes through the support.

A potentially promising method for nucleation, fixation, and formation of supramolecular objects on a substrate is physical vapor deposition (PVD), in which the molecular building blocks are deposited from the gas phase. Since PVD is a solvent‐free process, no solubilizing groups on the molecular building blocks are required. Under a proper set of conditions, similarly as described earlier, this process allows for the formation of nanostructures such as nanofibers or nanobelts.^[^ ^18^ ^]^ Several research groups have demonstrated the potential of PVD‐processed nanomaterials in optoelectronic applications^[^ ^19^ ^]^ including sensors^[^ ^20^ ^]^ and transistors^[^ ^21^ ^]^ as well as in biomedical and health care applications.^[^ ^22^ ^]^ Molecular building blocks, which were used for this purpose, are among others pentacene, perylene, and porphyrin derivatives. These aromatic compounds were selected for the preparation of nanomaterials by PVD because of their high thermal stability and evaporability as well as their ability to form aggregates via *π*–*π*‐interactions. In this context, a less investigated molecular building block is tricycloquinazoline (TCQ). TCQ is a nitrogen‐containing heteroaromatic compound with high chemical and thermal stability, which feature light absorption in the visible range up to about 500 nm.^[^ ^23^, ^24^ ^]^ TCQ derivatives has been investigated as discotic liquid crystals^[^ ^25^ ^]^ and more recently as covalent organic frameworks. The latter have gained attention due to their conductivity^[^ ^26^ ^]^ and gas sorption properties,^[^ ^27^ ^]^ which enabled the use as cathode materials in batteries^[^ ^28^ ^]^ and as electrocatalysts in CO_2_ reduction.^[^ ^29^ ^]^ However, the use of TCQ as a heterogeneous photocatalyst has not yet been demonstrated.

**Figure 1:**
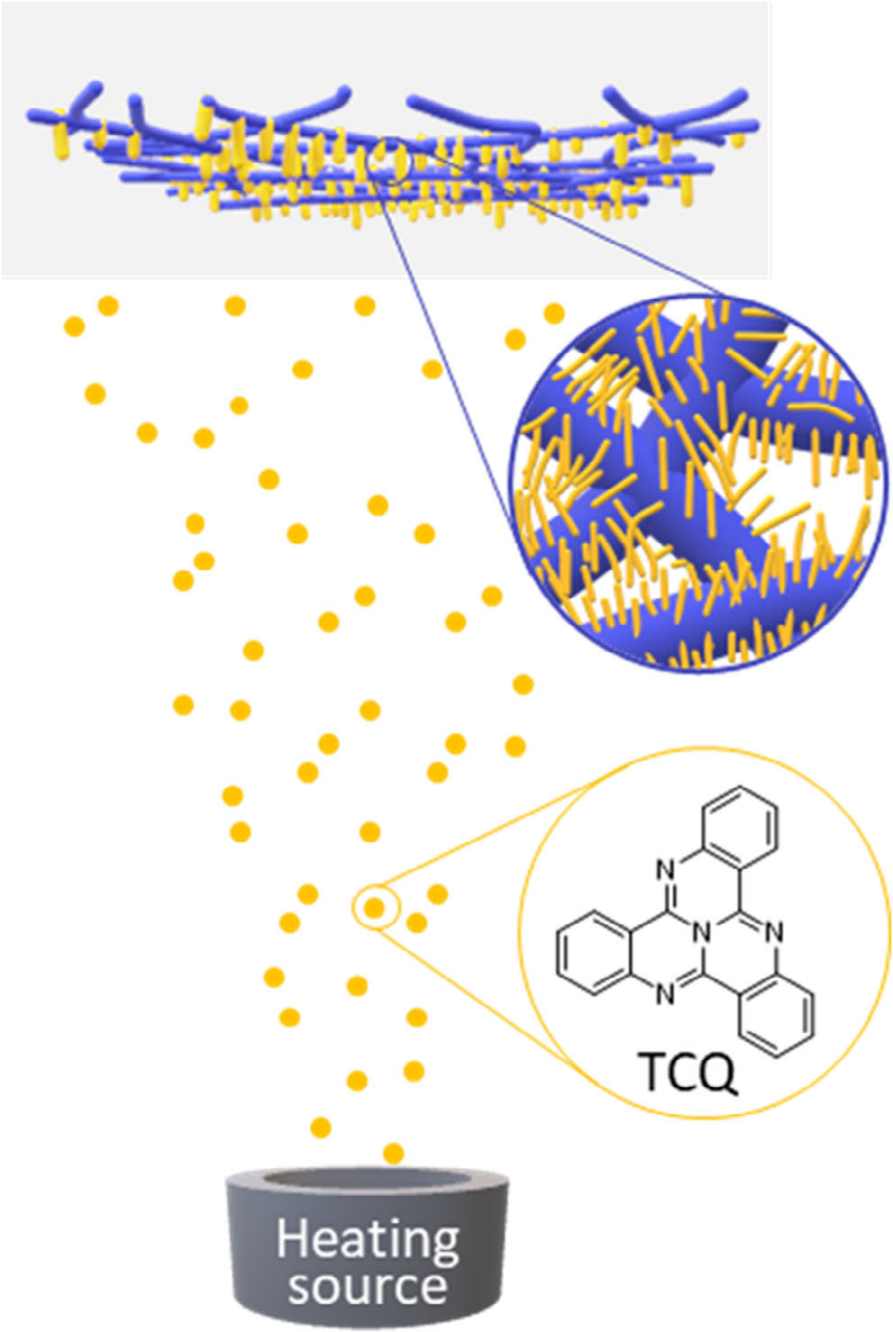
Schematic illustration of the physical vapor deposition (PVD) of tricycloquinazoline (TCQ, orange) forming supramolecular nanofibers on a microglass‐fiber support (blue) yielding mesostructured nonwovens with a bottlebrush‐like morphology for photocatalytic applications.

Here, we report on mesostructured nonwovens with supramolecular TCQ nanofibers, suitable as a heterogeneous photocatalyst for the photocatalytic degradation of organic pollutants such as rhodamine B and tetracycline. Such mesostructured nonwovens were realized by using PVD as a straightforward method to well‐defined supramolecular nanofibers of TCQ on a glass‐microfiber support (**Figure** **1**). To demonstrate control over the morphology of TCQ nanofiber layers by PVD on various compact substrates, we investigated several process parameters, including deposition time, substrate material, and substrate geometry. By using glass‐microfiber nonwovens as porous support, mesostructured nonwovens with a bottlebrush‐like morphology comprising supramolecular TCQ nanofibers were obtained. The morphology remains intact when used in a batch‐type photodegradation process of rhodamine B in aqueous media. Applying continuous flow conditions, the supramolecular TCQ nanofibers still remain attached on the glass‐fiber support without leaching into the reaction solution. Due to the fixation of TCQ nanofibers on the support, these functional mesostructured nonwovens can be used, removed, and reused demonstrating the potential of supported TCQ nanofibers as photocatalysts for the degradation of organic pollutants.

## 2 Results and Discussion

### 2.1 Preparation of Supramolecular Nanofibers via PVD

**Figure 2:**
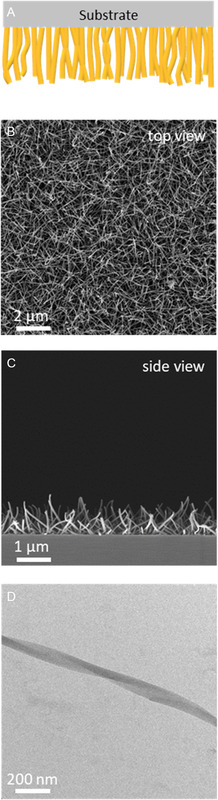
A) Schematic view of supramolecular TCQ nanofibers physical vapor deposited onto a silicon wafer as substrate. B,C) SEM images of the TCQ nanofibers: top view and side view. D) TEM image of an isolated TCQ nanofiber. PVD conditions: *T*_source_ = 200 °C, *p* = 10^−6^ mbar, deposition time: 300 s, and *T*_substrate_ = 25 °C.

TCQ is a C_3_‐symmetric heteroaromatic disclike compound, which can be obtained in a tricyclization reaction of three *o*‐aminobenzonitriles using catalytic amounts of ZnCl_2_ as described by Ponomarev et al.^[^ ^24^ ^]^ Complete conversion to the product can be monitored by Fourier‐transform infrared (FTIR) spectroscopy due to the absence of nitrile (CN) stretching vibrations at 2207 cm^−1^ and two amine (NH)‐stretching vibrations at 3366 and 3458 cm^−1^ (Section S2, Supporting Information). The molecular structure of the TCQ was clearly identified by analytical methods such as proton nuclear magnetic resonance (^1^H NMR) spectroscopy and mass spectrometry (MS). Differential scanning calorimetry (DSC) reveals a melting point *T*_melt_ for TCQ at 317 °C and a crystallization temperature of *T*_cryst_ at 293 °C (Section S3, Supporting Information). Thermogravimetric analysis (TGA) shows that TCQ starts to evaporate at 325 °C. This process is completed without residue at ≈420 °C. Thus, high purification can be easily performed by sublimation in high vacuum at about 300 °C resulting in fine yellow needles in a good yield. These findings already provide evidence that TCQ can be processed to fiber‐like structures, which is schematically depicted in **Figure** **2**A for the deposition on substrates in a controlled manner by PVD techniques. For this, we have used a custom‐made PVD chamber with an effusion cell source loaded with TCQ. As processing conditions, a reduced pressure of 10^−6^ mbar, 200 °C of source temperature, and a substrate temperature of 25 °C were selected. At these conditions, a constant evaporation rate was monitored by a quartz crystal microbalance during the entire processing time. For initial morphology investigations, TCQ was deposited for 300 s on a silica wafer. Scanning electron microscopy (SEM) investigations (Figure 2B,C) reveal that TCQ forms a homogeneously and densely packed layer of supramolecular nanofibers. In particular, the side‐view microscopy image indicates a grasslike morphology across the whole substrate with the supramolecular nanofibers growing in a perpendicular manner away from the substrate. Although we cannot clearly visualize the first deposited layers of TCQ, we attribute this dense fiber growth behavior to a large number of small TCQ nuclei initially formed on the substrate. At these conditions, the nanofiber diameter was determined to be 66 ± 11 nm featuring a nanofiber‐layer thickness of about 1.0 μm. Transmission electron microscopy (TEM) analysis of scratched off supramolecular TCQ nanoobjects indicate a ribbonlike and partially twisted structure with a typical width of about 70 nm (Figure 2D), which we refer in the following section as nanofibers. Selected‐area electron diffraction (SAED) on these nanofibers demonstrates that TCQ is highly oriented along the fiber's long axis (Section S4, Supporting Information). Although the SAED cannot rule out some degree of disorder, DSC measurements using these nanofibers shows only a single melting point upon first heating indicating one polymorph. From these data, a TCQ inter‐disc distance of 3.9 Å (d‐spacing in the longitudinal direction) and TCQ intercolumnar distance of 9.7 Å (d‐spacing in the perpendicular direction) can be determined. These findings are in good agreement with the inter‐disc and intercolumnar stacking as found in the crystal structure of TCQ.^[^ ^30^ ^]^

**Figure 3:**
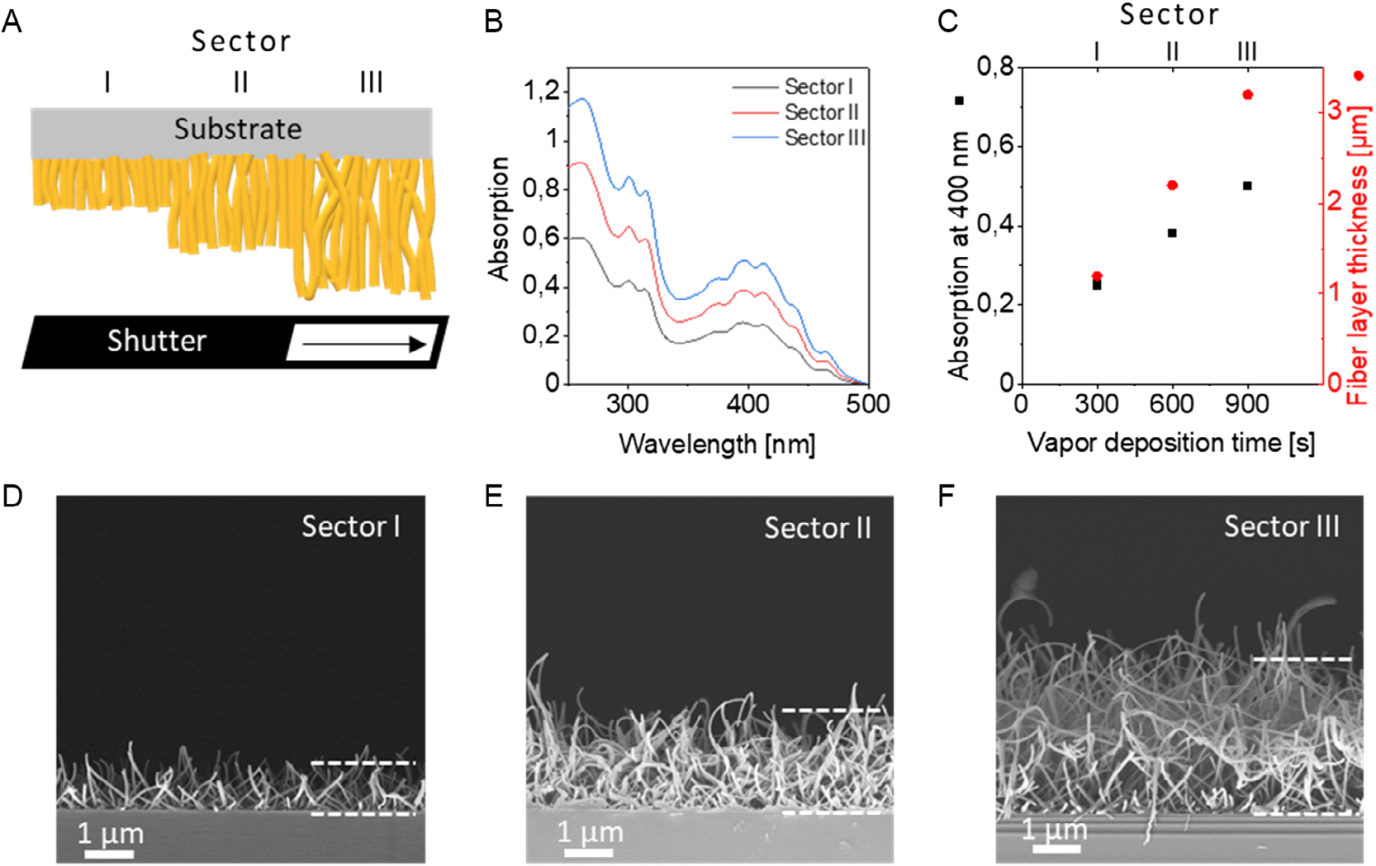
A) Schematic illustration of supramolecular TCQ nanofibers with increasing fiber‐layer thicknesses by combinatorial vapor deposition with a shutter being moved stepwise from left to right. The vapor deposition time for sector I is 300 s, for sector II 600 s, and for sector III 900 s. B) Absorption spectra of sector I (black), II (red), and III (blue) on a quartz glass substrate. C) Absorption at 400 nm (in black) and the TCQ fiber‐layer thickness (in red) as function of the vapor deposition time revealing an average fiber growth rate of 3.3 nm s^−1^. D–F) Side‐view SEM images of TCQ nanofibers for sectors I, II, and III on a silicon wafer with a fiber‐layer thickness of about 1.0, 2.1, and 3.1 μm. The dotted lines are a guide to the eye to indicate the thickness. PVD conditions: *T*_source_ = 200 °C, *p* = 10^−6^ mbar, and *T*_substrate_ = 25 °C.

To investigate how the fiber density and fiber length can be controlled by the evaporation time, we performed a combinatorial experiment with three sectors I–III as schematically depicted in **Figure** **3**A. By moving a shutter by one third of the substrate width every 300 s during vapor deposition, we established a stepwise gradient of the fiber‐layer height on the same substrate. A silicon wafer substrate was used for SEM investigations and a quartz glass substrate for absorption measurements. TCQ was continuously deposited on both substrates at 10^−6^ mbar and 200 °C source temperature with sectors I–III being exposed to the vapor for 300, 600, and 900 s.

Prior to the optical properties of the nanofibers, we determined the absorption spectra of TCQ in CHCl_3_ solution (Section S5, Supporting Information). Molecularly dissolved TCQ features a structured absorption with the maximum of the 0–0 transition being at 450 nm. The absorption spectra on nanofibers show similar features, yet the absorption is slightly broadened and the 0–0 transition is redshifted by about 15 nm, which is attributed to aggregation. Very similar spectra were found when performing UV–vis spectroscopy on the different sectors of the quartz glass slide (Figure 3B). With increasing deposition time of TCQ, the absorbance is increasing. Figure 3C indicates a linear correlation of the deposition time and the absorbance in the sectors I–III. From the glass slides, we determined the optical gap of the TCQ nanofibers derived from a Tauc plot of the UV–vis spectrum to be 2.57 eV (Figure S6, Supporting Information).

SEM side‐view images of TCQ supramolecular nanofibers on the silicon substrate show a similar trend, namely an increase in the fiber‐layer thickness (Figure 3D–F). Notably, the homogeneity and density of the nanofibers within each section are highly comparable and the TCQ nanofiber diameter of about 70 nm remains constant. We found that the nanofiber‐layer thickness for the sectors I–III increases steadily from 1.0 to 2.1 to 3.1 μm demonstrating a high control over the resulting supramolecular nanofiber morphology. To validate that the morphology of the TCQ nanofibers is comparable on different substrate types, we investigated by SEM the nanofiber morphology on glass, quartz glass, and plasma‐etched glass slides as well as on poly(lactic acid) films. For all tested types of substrates, the morphology of the TCQ nanofibers is highly identical (Section S7, Supporting Information). This finding suggests that a very comparable kind and number of nuclei are present on the different substrates at a substrate temperature of 25 °C initiating the nanofiber growth.

### 2.2 Preparation of Supramolecular TCQ Nanofiber/Glass‐Microfiber Nonwovens

**Figure 4:**
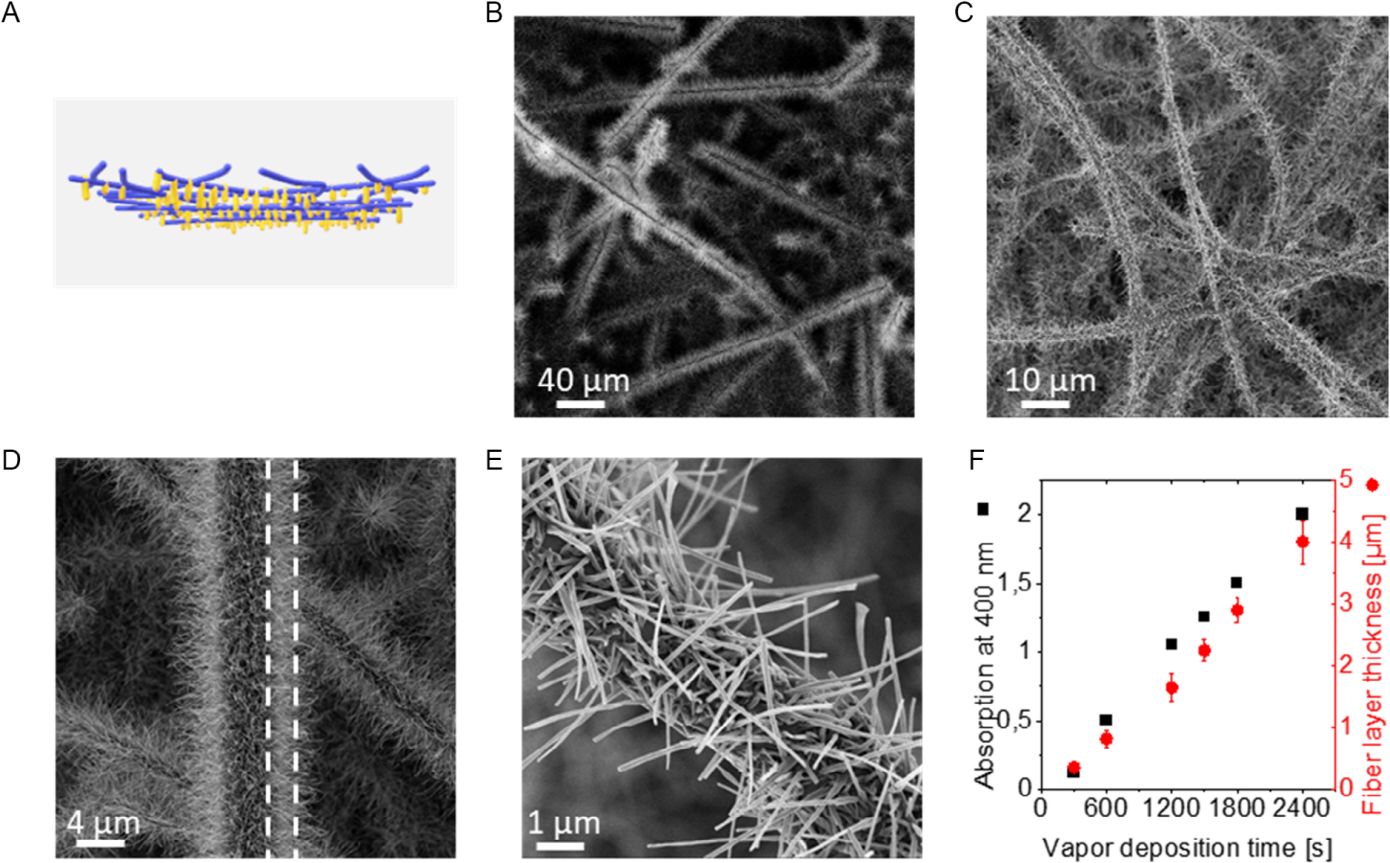
A) Schematic illustration of the supramolecular TCQ nanofiber/glass‐microfiber nonwovens prepared by PVD. B,C) SEM overview image of a mesostructured nonwoven after a vapor deposition time of 1500 s. D,E) SEM images of the same nonwoven at different magnifications showing a bottlebrush‐like morphology. The dashed lines in (C) indicate the fiber‐layer thickness of about 2 μm surrounding the glass microfiber. F) Absorption at 400 nm of TCQ nanofibers on glass slides (in black) as function of the vapor deposition time and TCQ fiber‐layer thickness (in red) at identical conditions. The calculated TCQ nanofiber growth rate was found to be 1.7 nm s^−1^. PVD conditions: *T*_source_ = 200 °C, *T*_substrate_ = 25 °C, *p* = 10^−6^ mbar, and deposition time = 1500 s.

To make use of the supramolecular TCQ nanofibers as photoactive material in aqueous media, we deposited the nanofibers on a porous support to achieve a supramolecular TCQ nanofiber/glass‐microfiber nonwoven. Such mesostructured nonwovens are beneficial, because they can be used under batch and continuous flow conditions, where the immobilized supramolecular TCQ nanofibers can be easily placed into water, removed, and reused again. Since the morphology of the deposited supramolecular TCQ nanofibers is highly independent on the substrate material, we have selected a commercial glass‐microfiber nonwoven as porous support. The round‐shaped glass‐fiber nonwoven has a diameter of 37 mm and a thickness of 0.4 mm. It features a distribution of submicron and microfibers with an average fiber diameter of 2.2 ± 1.7 μm (Section S8, Supporting Information) indicating that the nonwoven consists of some hundred layers of glass microfibers. Ultimately, supramolecular TCQ nanofiber/glass‐microfiber nonwoven were prepared as schematically depicted in **Figure** **4**A by PVD employing the same conditions as described before. Figure 4B,C shows SEM images of the supramolecular TCQ nanofiber/glass‐microfiber nonwoven obtained after 1500 s of deposition time at different magnifications. The top view images reveal that the glass‐fiber nonwoven is entirely and homogenously covered with supramolecular TCQ nanofibers. They also show that several layers of the glass microfibers are covered indicating a larger accessible area for the TCQ vapor stream. At larger magnifications (Figure 4D,E), it can be observed that supramolecular TCQ nanofibers have been grown away from the support microfibers in a similar manner as found after deposition of TCQ on glass slides. The nanofiber lengths and diameter are well defined indicating a high control over these parameters during processing. The average TCQ nanofiber diameter is 75 nm, being in good agreement as found in the previous PVD experiments with the glass slides. Interestingly, the magnification also indicates that TCQ deposits not only on top of the glass microfibers, but also around the glass microfibers resulting in a bottlebrush‐like morphology. The TCQ fiber‐layer thickness after 1500 s of evaporation is about 2 μm, which is significantly thinner compared to glass slides and attributed to the larger accessible surface area. To get a clearer picture with respect to the TCQ nanofiber growth rate in the glass‐fiber nonwovens, we have prepared a series of mesostructured nonwovens by varying the deposition time of 300, 600, 1200, 1500, 1800, and 2400 s and investigated the specimens by SEM (Section S9, Supporting Information). The microscopy images show that nanofiber growth is initiated simultaneously and homogeneously at the entire accessible surface area of the glass‐fiber nonwoven. We also found that the nanofiber diameter in all mesostructured nonwovens is almost the same indicating that only the nanofiber length is varied with increasing evaporation time. The insets in Figure S9, Supporting Information (Section S9, Supporting Information), also show that the yellowish color on top of the mesostructured nonwovens is increasing with evaporation, whereas the backside of specimen is unchanged. Since UV–vis spectroscopy on the TCQ on the glass‐fiber nonwovens is not unambiguously possible due to strong scattering of the nonwoven, we have additionally placed a quartz glass slide next to the glass‐fiber nonwoven in the same substrate holder during the PVD experiment. This reference was used to determine the absorbance of the TCQ by UV–vis spectroscopy. Figure 4F shows the progress of the absorbance at 400 nm with increasing evaporation times determined from the quartz glass slides. The linear increase of the absorbance goes very well in line as found before indicating that the same amount of TCQ is deposited on the support. Similarly, the fiber‐layer thicknesses was found to increase linearly with evaporation time, however, with a significantly smaller fiber‐layer thickness as found before due to the large accessible surface area. As a result, the determined fiber growth rate of the TCQ nanofibers on the microfiber‐glass nonwoven was found to be of 1.7 nm s^−1^, which is approximately half the value.

### 2.3 Photocatalytic Degradation of Organic Pollutants

The potential of the TCQ nanofiber/glass‐microfiber nonwovens as heterogenous photocatalyst was studied for the degradation of organic pollutants in water. As organic pollutants, we focused on two compounds, namely rhodamine B and tetracycline. Rhodamine B is a widely used dye, which was selected, since the degradation process can be conveniently investigated by UV–vis. Moreover, the degradation pathways are literature‐known.^[^ ^31^ ^]^ The second compound, tetracycline, is a widely used broad‐spectrum antibiotic.^[^ ^32^ ^]^ Tetracycline is difficult to metabolize in the human and animal digestive systems and therefore has the potential to accumulate in wastewater.^[^ ^32^, ^33^ ^]^ A prerequisite for the use of phototactically active organic materials is a high photochemical stability against the applied conditions during operation. To validate the suitability of the TCQ nanofiber/glass‐microfiber nonwovens, we have conducted UV light exposure tests under harsh conditions. For this, a mesostructured nonwoven was immersed in deionized water and the upper side with the TCQ nanofibers was exposed to 5 suns of UV radiation for 48 h. After removing and drying, the TCQ were fully dissolved and analyzed by high‐pressure liquid chromatography (HPLC). HPLC elugrams of solutions before and after UV light exposure reveal only signals of TCQ (Section S10, Supporting Information). Moreover, HPLC analysis of the residual water shows that no further organic compounds can be found demonstrating the high photochemical stability of TCQ under these conditions. Further evidence is provided by SEM investigations before and after the UV exposure test showing that the bottlebrush‐like morphology remains intact (Section S11, Supporting Information).

In a first set of experiments, we investigated the potential of the TCQ nanofiber/glass‐microfiber nonwovens as a photocatalyst in a batch‐type process. The setup is shown in Section S12, Supporting Information. As a light source, we used a 6200 K light‐emitting diode (LED) with an irradiance of 29 mW cm^−2^ in the visible‐light regime ranging from 400–700 nm, which fits to the TCQ absorption (Section S12, Supporting Information). The prepared and used mesostructured glass‐fiber nonwoven with a diameter of 37 mm and a TCQ nanofiber length of 4 μm as described earlier. These nonwovens contain in total 0.4 mg of TCQ nanofibers resulting in a loading capacity of TCQ nanofibers of 0.05 mg cm^−2^. The mesostructured nonwoven is placed in the reactor setup in a manner, where TCQ nanofibers are facing to the light source. A 10^−5^ mol L^−1^ rhodamine B solution in water was used since this concentration can be conveniently monitored via UV–vis spectroscopy. The progress of the decomposition was recorded in 15 min intervals for 4 h. The UV–vis spectra show that during degradation the absorption maximum shifts from 554 to 500 nm (Section S13, Supporting Information). This behavior is attributed to a degradation pathway via *N*‐deethylation of rhodamine B.^[^ ^31^ ^]^ The UV–vis spectra also reveal a rapid decomposition of rhodamine B (Section S13, Supporting Information) with 92% of the rhodamine B being decomposed within 4 h. This type of reaction requires a permanent illumination forming the reactive species for degradation, which quickly stops when the illumination is turned off. This is demonstrated by applying successive 30 min light‐on and light‐off cycles for the degradation of rhodamine B in the presence of a mesostructured nonwoven for 7.5 h in total (see Section S14, Supporting Information). In the “dark,” the reaction largely comes on halt, yielding eventually almost the same result as obtained for the permanent illumination. Assuming a pseudo first‐order kinetic for the reaction using permanent illumination, the apparent rate constant *k*’ can be calculated by ln(*c*/*c*_0_) = *k*′t and was determined to 1.0 × 10^−2^ min^−1^. SEM investigations on mesostructured nonwovens after photocatalysis shows that the bottlebrush‐like morphology is maintained (Section S15, Supporting Information).

To investigate how the immobilization of TCQ nanofibers on the nonwoven fabric affects the photocatalytic activity, a reference experiment was conducted using only unsupported TCQ nanofibers as photocatalyst. For this, the same amount, namely 0.4 mg of TCQ nanofibers, was added to the reaction chamber. The photocatalytic degradation of rhodamine B using immobilized TCQ and unsupported TCQ is shown in Section S16, Supporting Information. Interestingly, the photocatalytic degradation progresses faster with the mesostructured nonwoven. The corresponding rate constant *k*’ for the experiment with the unsupported TCQ nanofibers is 7.8 × 10^−3^ min^−1^, which corresponds to a 22% decrease compared to the immobilized TCQ nanofibers. This lower activity can be attributed to agglomeration of the TCQ nanofibers and thus a decrease in the active surface area.

To elucidate the mechanism of photocatalytic degradation of organic pollutants by TCQ, we studied the photodegradation of rhodamine B in the presence of various scavengers capable of scavenging specific reactive oxygen species during the reaction. As radical scavengers, we used isopropanol (^*i*^PrOH), *p*‐benzoquinone (*p*‐BQ), and triethanolamine (TEOA) for hydroxyl and superoxide radicals, as well as holes, respectively.^[^ ^34^ ^]^ The influence of the different scavenger molecules on the photocatalytic degradation of an aqueous rhodamine B solution using a mesostructured nonwoven in a batch‐type setup is depicted in Section S17, Supporting Information. The use of ^*i*^PrOH as a scavenger shows no negative effect on photocatalysis, suggesting that hydroxy radicals are not involved in the degradation of rhodamine B. The degradation rate *k*’ in the presence of TEOA was found to be 25% slower than compared to the reaction where no scavenger is present. This indicates that photogenerated holes may play a role as reactive species in the degradation. The strongest influence on the reaction rate, however, was observed with *p*‐BQ as scavenger. Here, the rate constant decreased 85% of the original value, indicating the superoxide radicals generated via reduction of O_2_ by photogenerated electrons are the main reactive species for the degradation of rhodamine B.

In contrast to batch‐type processes, processes under continuous flow conditions have the advantage that larger quantities can be converted and a lower treatment time may be required.^[^ ^35^ ^]^ Thus, we investigated the suitability of TCQ nanofiber/glass‐microfiber nonwovens as photocatalyst for degradation of the dye rhodamine B and the common antibiotic tetracycline in water under continuous flow conditions.

**Figure 5:**
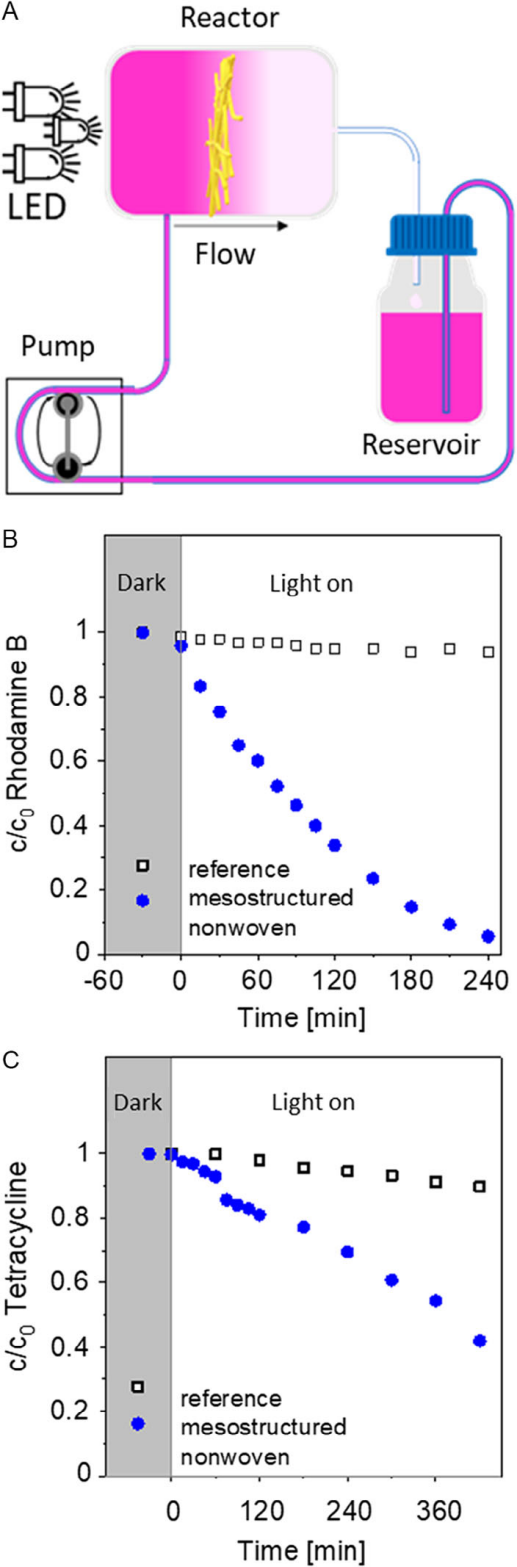
A) Schematic illustration of photocatalytic degradation of organic pollutants using a continuous flow reactor: Organic pollutants are circulated from a reservoir solution with a flow rate of 1.5 L h^−1^. The nanofiber/microfiber nonwoven is fixated as a heterogenous photocatalyst upon visible‐light LED irradiation. B) Progress of photodegradation of rhodamine B as a function of time without (dark) and with irradiation (light on) using mesostructured nonwovens (closed circle) and neat glass microfibers as reference (open squared). C) Progress of photodegradation of tetracycline as a function of time without (dark) and with irradiation (light on) using nanofiber/microfiber nonwovens and neat glass microfibers as reference.

A schematic illustration of the circulating continuous flow photoreactor is shown in **Figure** **5**A and a photograph in Section S12, Supporting Information. For all experiments, mesostructured nonwovens as described earlier for the batch‐type photodegradation were prepared. Similarly, the mesostructured nonwoven was clamped in the photoreactor chamber with the TCQ nanofibers facing the light source. The tenfold volume of an aqueous solution of the dye rhodamine B with the same initial concentration of 10^−5^ mol L^−1^ was circulated with a peristaltic pump through the photoreactor. The degradation pathway of rhodamine B using a TCQ nanofiber/glass‐microfiber nonwoven under these conditions proceeds as shown before (see Section S18, Supporting Information) and 95% of the initial concentration was degraded within 4 h as shown in Figure 5B. This value is very close to that found for the batch‐type reaction. The apparent rate constant *k*’ with the mesostructured nonwoven was calculated to be 1.1 × 10^−2^ min^−1^. As reference, a photolysis experiment, that is illumination without photocatalyst, was performed. Using a rhodamine B solution with the same concentration, we found that only minor portion of 5% was degraded within 4 h. The apparent rate constant *k*’ of the photolysis was found to be 9.05 × 10^−4^ min^−1^, which is a reduction by a factor of 12 demonstrating only a minor degradation without the mesostructured nonwoven.

To evaluate how much of the degraded rhodamine B was mineralized to CO_2_, we performed total organic carbon (TOC) measurements after the photocatalysis using the TCQ nanofiber/glass‐microfiber nonwoven. The TOC value for the initial rhodamine B solution is 4.26 mg L^−1^ and drops after photocatalysis to 2.8 mg L^−1^, which means that 35% are decomposed to CO_2_, while the remaining 65% are decomposed to other products.

After photodegradation experiments, we investigated the remaining water solution toward traces of TCQ by HPLC applying appropriate conditions (Section S19, Supporting Information) and found that no TCQ leaches from the mesostructured nonwoven after 4 h of circulation. This encouraged us to investigate the reusability of the same mesostructured nonwovens as photocatalysts by performing three successive runs using freshly prepared rhodamine B solutions for each run. For all runs, no significant loss in the photocatalytic activity was observed (Section S20, Supporting Information) indicating that the TCQ nanofibers remains on the glass‐fiber nonwoven. SEM investigations of these mesostructured nonwovens after the third run reveal that although the initial bottlebrush‐like structure is not clearly maintained, indeed the supramolecular TCQ nanofibers are still on top of the glass‐microfiber nonwoven (Section S21, Supporting Information) and contribute in the same manner to the photocatalytic degradation without detaching and leaching from the glass‐microfiber nonwoven. We also analyzed the photocatalyst after the reusability test by HPLC measurements to investigate if changes in the molecular structure of the TCQ occur after use. The HPLC elugrams of the photocatalyst dissolved from the mesostructured nonwoven and TCQ as reference, both elugrams show only one peak, demonstrating the high chemical stability of TCQ (see Section S22, Supporting Information).

Finally, we have investigated the supramolecular TCQ nanofiber/glass‐microfiber nonwovens for the photocatalytic degradation of tetracycline, an antibiotic that often can be found in waste water. Here, we have used an aqueous solution of tetracycline with an initial concentration of 10^−4^ mol L^−1^. All other parameters with respect to setup of the continuous flow photoreactor, illumination procedure, and the composition and size of the mesostructured nonwoven were used as described earlier. In contrast to the experiments with rhodamine B, the progress of the tetracycline degradation is conducted by HPLC (Section S23, Supporting Information). As shown in Figure 5C, circulation in the dark in the presence of the mesostructured nonwoven shows no change. Similarly, photolysis, without the photocatalysts, shows only a minor decomposition of tetracycline. Under illumination of the TCQ‐mesostructured nonwoven with 0.05 mg cm^−2^ loading, the tetracycline is degraded by 60% within 7 h. Determination of the amount of TOC in the remaining solution revealed that 40% of the degraded tetracycline concentration was mineralized to CO_2_.

## 3 Conclusion

We have demonstrated a straightforward method to fabricate supramolecular nanofibers based on TCQ by PVD into dense and homogeneous fiber layers with full control over the fiber length by adjusting the deposition time. At these conditions, we found a uniform nanofiber diameter of about 70 nm, which can also be realized on different substrates. Utilizing glass‐microfiber nonwoven as porous support material allowed us to obtain TCQ nanofiber/glass‐microfiber nonwovens with a bottlebrush‐like morphology. We showed that these mesostructured nonwovens act as photocatalysts in the visible light for the degradation of the dye rhodamine B and the antibiotic tetracycline under continuous flow conditions in aqueous solutions. These findings may pave the way to realize novel and robust functional supramolecular nanostructures with controlled morphology via an elegant PVD‐processing method, which can be used and reused in catalytic applications.

## 4 Experimental Section

4.1

4.1.1

##### Materials

For the synthesis and characterization of TCQ, see Section S1, Supporting Information. Glass‐microfiber filter (37 mm, MN85/90BF) was purchased by Macherey‐Nagel GmbH & Co. KG. Rhodamine B was purchased by Kremer Pigmente GmbH & Co. KG and tetracycline Hydrochloride from Carl Roth GmbH & Co. KG. Isopropyl alcohol and benzoquinone were purchased from Merck KGaA. TEOAs were purchased from abcr GmbH.

##### Methods

UV–vis absorbance spectra were recorded on a Jasco V‐670 spectrophotometer. SEM was performed with a Zeiss 1530 field‐emission SEM (FESEM) at 3 kV using Inlens detector. Samples were fixed via a double‐sided adhesive conductive carbon tape on an SEM sample holder and subsequently sputtered with platinum (2 nm) prior SEM investigation.

TEM was performed on a JEOL JEM‐2200FS at 200 kV. Samples were prepared by drop‐casting a suspension of the powder onto a carbon‐coated copper grid (S160, Plano EM, Germany) and the supernatant solution was removed by a filter paper. The specimens were imaged at room temperature in bright‐field and SAED mode using a Gatan OneView camera. TOC measurements were performed on a TOC‐L analyzer (Shimadzu, Kyoto, Japan) by using the 680 °C combustion catalytic oxidation method. Carbon content could be measured in concentrations from 4 μg L^−1^ to 30.000 mg L^−1^. Total sample combustion was achieved by heating to 680 °C in an oxygen‐rich environment inside combustion tubes filled with a platinum catalyst. The generated CO_2_ was detected by an infrared gas analyzer.

##### Physical Vapor Deposition of TCQ

For preparation of nanofiber layer of TCQ, a modified vapor deposition chamber PLS 500 from Balzers was used. Quartz crystal sensors were mounted near the substrate and used to monitor the evaporation rate. About 300 mg of TCQ was weighed into a quartz crucible, which was placed into an effusion cell (heating source). At 10^−6^ mbar and 200 °C temperature of the effusion cell, a constant apparent evaporation rate was monitored by the quartz crystal sensors and found to be 3.3 nm s^−1^. The shutter was fully opened to start the deposition onto the substrate. At the end of the experiment, the vacuum chamber was ventilated with air. For the preparation of step thickness gradients, a combinatorial setup was used to obtain different sectors.^[^ ^36^ ^]^ The substrate holder was a custom‐made 3D‐printed holder with cavities either for three quartz slides (76.2 × 25.4 × 1.0 mm) or for 2 × 1 glass‐fiber filters with a diameter of 37 mm and a cavity for a glass slide for reference measurements.

##### Photocatalytical Degradation Experiments

For initial photocatalytical experiments, the degradation of rhodamine B in a batch setup was conducted. For this, a mesostructured nonwoven (TCQ loading of 0.4 mg or 0.05 mg cm^−2^) was placed in a batch reactor and aqueous rhodamine B solution (0.02 L, 10^−5^ mol L^−1^) were added. After every 15 min of irradiation with 6200 K LED (29 mW cm^−2^), the rhodamine B concentration was determined photometrically by UV–vis. As reference experiment, 0.4 mg TCQ nanofibers were dispersed in aqueous rhodamine B solution (0.02 L, 10^−5^ mol L^−1^) in a batch reactor. Evaluation of the photocatalytic activity was conducted as described earlier. To investigate which reactive oxygen species is involved during the photodegradation to a solution of rhodamine B (0.02 L, 10^−5^ mol L^−1^), a mesostructured nonwoven (TCQ loading of 0.4 mg or 0.05 mg cm^−2^) and a scavenging agent (0.1 mL of TEOA or 0.1 mL of ^*i*^PrOH or 1.0 mg of *p*‐BQ) were mixed into the batch reactor. The photocatalytic reactions were subsequently performed as described earlier.

For the light on/off reference experiments, a solution of rhodamine B (0.02 L, 10^−5^ mol L^−1^) and a mesostructured nonwoven (TCQ loading of 0.4 mg or 0.05 mg cm^−2^) were brought together into the batch reactor. Light on/off cycles were performed in successive 30 min intervals turning the light on and off for a total time of 7.5 h.

In addition, a continuous flow reactor was used to study the photocatalytic activity under flow conditions. For this, an aqueous rhodamine B solution (0.25 L, 10^−5^ mol L^−1^) or an aqueous solution of tetracycline (0.25 L, 10^−4^ mol L^−1^) was circulated through the reactor with a volume of 0.1 L and a flow rate of 1.5 L h^−1^. The remaining volume ensured a constant continuous circulation of the solution. After every 15 min of irradiation with 6200 K LED (29 mW cm^−2^), the rhodamine B concentration was determined photometrically by UV–vis and the tetracycline concentration was determined using HPLC.

## Conflict of Interest

The authors declare no conflict of interest.

## Supporting information

Supplementary Material

## Data Availability

The data that support the findings of this study are available from the corresponding author upon reasonable request.
