# Supplementary material for: Mesostructured Nonwovens with Supramolecular Tricycloquinazoline Nanofibers as Heterogenous Photocatalyst
Source: Small Sci. 2023 Dec 10;4(2):2300160. doi: 10.1002/smsc.202300160 (PMC11935285; doi:10.1002/smsc.202300160)
Supplement: Supplementary file 1 — Supplementary Material [file SMSC-4-2300160-s001.pdf]

Supporting Information

Mesostructured Nonwovens with Supramolecular Tricycloquinazoline Nanofibers as Heterogenous Photocatalyst

*Dennis Schröder, Christian Neuber, Ulrich Mansfeld, Klaus Kreger, and Hans-Werner Schmidt**

Table of content

S1: Materials and methods

S2: Synthetic scheme, procedure, and molecular characterization of TCQ

S3: Thermal characterization of TCQ

S4: TEM micrograph and SAED of supramolecular TCQ nanofibers via physical vapor deposition

S5: UV-vis characterization of TCQ in solution and TCQ nanofibers prepared by physical vapor deposition

S6: Optical gap of supramolecular TCQ nanofibers

S7: Morphology investigations of physical vapor deposited TCQ nanofibers on different substrates

S8: Photo- and micrograph of the used commercial glass microfiber nonwoven

S9: Morphology investigations of supramolecular TCQ nanofiber/glass microfiber nonwovens

S10: HPLC spectra before and after UV/light exposure tests of supramolecular TCQ nanofiber/glass microfiber nonwovens

S11: SEM images before and after UV/light exposure tests of supramolecular TCQ nanofiber/glass microfiber nonwovens

S12: Setups for photocatalytic degradation of Rhodamine B using mesostructured nonwoven

S13: Photocatalytic degradation of Rhodamine B using mesostructured nonwoven in a batch setup

S14: Light on/off experiment during the photocatalytic degradation of Rhodamine B using mesostructured nonwoven in a batch setup

S15: Morphology investigations of supramolecular nanofiber/ glass microfiber nonwoven after photocatalysis in a batch setup

S16: Photocatalytic degradation of Rhodamine B using unsupported TCQ nanofibers in a batch setup

S17: Reaction mechanism studies on the photocatalytic degradation of Rhodamine B using mesostructured nonwoven in a batch setup

S18: Photocatalytic degradation of Rhodamine B using mesostructured nonwoven in a continuous flow setup

S19: Leaching test of TCQ from the mesostructured nonwoven

S20: Reusability test of supramolecular nanofiber/glass microfiber nonwoven

S21: Morphology investigations of supramolecular nanofiber/ glass microfiber nonwoven after photocatalysis

S22: HPLC investigations of supramolecular nanofiber/glass microfiber nonwoven after photocatalysis

S23: Photocatalytic degradation of Tetracycline using a continuous flow reactor

S1: Materials and Methods

Materials:

o-Aminobenzonitrile (98%) was purchased by Fluka Analytical. Anhydrous zinc chloride (98%) was purchased from Grüssing GmbH. α-Chloronaphthalene and diethyl ether was received by Sigma Aldrich and were used as received.

Methods:

^{1}H NMR and ^{13}C NMR spectra in solution were recorded on a Bruker Avance Ultrashield 300 (300 MHz and 75 MHz, respectively) at room temperature. For the preparation of the NMR samples, approx. 5 mg of the compounds were dissolved in 0.6 mL of $CDCl_3$.

High resolution mass spectra (HRMS) were carried out on a FINNIGAN MAT 8500 spectrometer from Thermo-Fisher Scientific using electron spray ionization (ESI).

Infrared (IR) spectra were recorded using a small amount of the solid powder on a PerkinElmer Spectrum 100 FT-IR spectrometer in attenuated total reflection (ATR) mode in the range from 4000 cm^{-1} to 650 cm^{-1}.

Thermogravimetric analyses (TGA) were performed in the range from 30 to 700 °C with a heating rate of 10 K min^{-1} under a nitrogen atmosphere with a flow rate of 30 mL min^{-1} using a Mettler Toledo TGA/DSC3+ Star.

Melting points and recrystallisation were investigated by differential scanning calorimetry using a Mettler Toledo DSC2. Approximately 10 mg of the solid were filled in sealable crucibles. Spectra were recorded with a heating/cooling rate of 10 $K\cdot min^{-1}$ in a temperature range from 30 °C to 330 °C.

Elemental analysis was measured with a Vario El III instrument from Elementar Analysen-Systeme.

To test the photochemical resistance and reveal potential degradation of tricycloquinazoline (TCQ) nanofibers against strong light irradiation, we have performed a UV/light exposure test. For this, supramolecular TCQ nanofiber/glass microfiber nonwovens were irradiated under controlled accelerated weathering conditions using a Q-SUN XE-3 test chamber (Q-LAB Corporation, Westlake, OH). Three xenon arc lamps equipped with a Daylight-Q filter system were used as irradiation source. The irradiance was set to 60 W/m^2 at 300–400 nm and performed at a relative humidity of 50%. The total irradiance was 594 W/m^2 resembling approximately the 5-fold of standard outdoor irradiation.

High performance liquid chromatography of TCQ was performed on an Agilent 1100 with a C_{18} reversed phase stationary phase with an eluent of acetonitrile/water 70/30 with of low rate of 1mL min^{-1} and an UV-detector. The injection volume was 10 µL. Retention time of TCQ was determined to be 20.1 min.

High performance liquid chromatography of tetracycline was performed on an Agilent 1260 Infinity II with a Zorbax SB-C18 stationary phase with an eluent of water (1% formic acid) /MeOH 85/15 with a flow rate of 1mL min^{-1} and an UV-detector. The injection volume was 5 µL. Retention time of tetracycline was determined to be 5.85 min.

S2: Synthetic scheme, procedure, and molecular characterization of TCQ

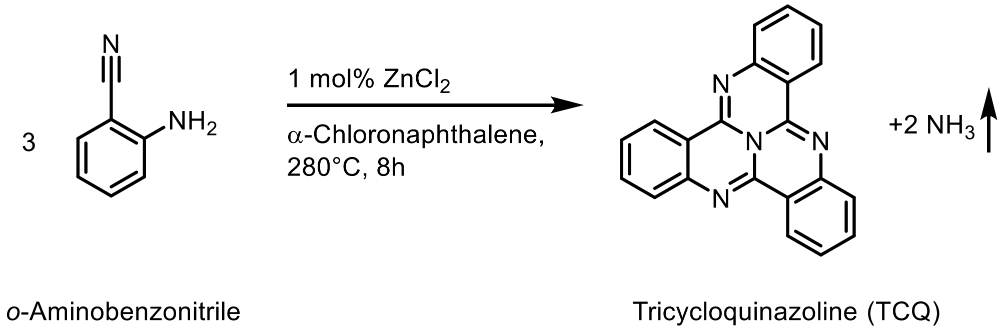

Figure S1. Synthetic scheme to TCQ as described by Ponomarev *et al.*.[1]

5.9 g (50 mmol, 1 eq.) of *o*-aminobenzonitrile and 68 mg (0.5 mmol, 0.01 eq.) of anhydrous zinc chloride was heated in 30 mL of α-chloronaphthalene in argon atmosphere at 290 °C for 8 h. Upon cooling to room temperature, a precipitate was obtained and filtered off. The solid was washed three times with 50 mL of diethyl ether and subsequently dried in vacuo. The crude product was purified by sublimation at 10^{-6} mbar and 300 °C to give 4.5 g (14 mmol, 84 % yield) of TCQ as fine yellow needles.

^{1}H NMR (300 MHz, $CDCl_3$, δ): 8.47 (dd, *J* = 8.1, 1.2 Hz, 3H), 7.61 (ddd, J = 8.1 7.1, 1.5 Hz, 3H), 7.52 (dd, J = 8.1, 1.3 Hz, 3H), 7.35 (ddd, J = 8.1, 7.1, 1.3 Hz, 3H)) ppm.

^{13}C NMR (75 MHz, $CDCl_3$, δ): 145.73, 144.78, 134.00, 127.07, 126.96, 126.26, 121.03 ppm.
HRMS (ESI) *m*/*z*: $[M + H]^+$ calcd for $C_{21}H_{12}N_4$, 321.1135; found, 321.1135.

Anal. calcd for $C_{21}H_{12}N_4$: C 78.73, H 3.78, N 17.49; found: C 77.29, H 4.01, N 16.98.

FT–IR: ν = 3061 (w), 1619 (s), 1590 (s), 1565 (s), 1476 (s), 1459 (m), 1335 (m), 1297 (m), 1234 (m), 1160 (m), 1136 (s), 1026 (m), 923 (m), 754 (s), 694 (s), 676 (s), 594 cm^{-1} (m).

Mp (DSC,second heating): 317 °C

UV–vis (*chloroform*): λ_{max} (ε) = 296 (22000), 309 nm (18000), 380 (17700), 401 (16800), 426 (6300), 453 (2340).

[1] Ponomarev, II; Vinogradova, S. V. An Efficient Method for the Synthesis of Tricycloquinazoline. *Bull. Acad. Sci. USSR, Div. Chem. Sci. (Engl. Transl.)* **1990**, *39*, 2229.

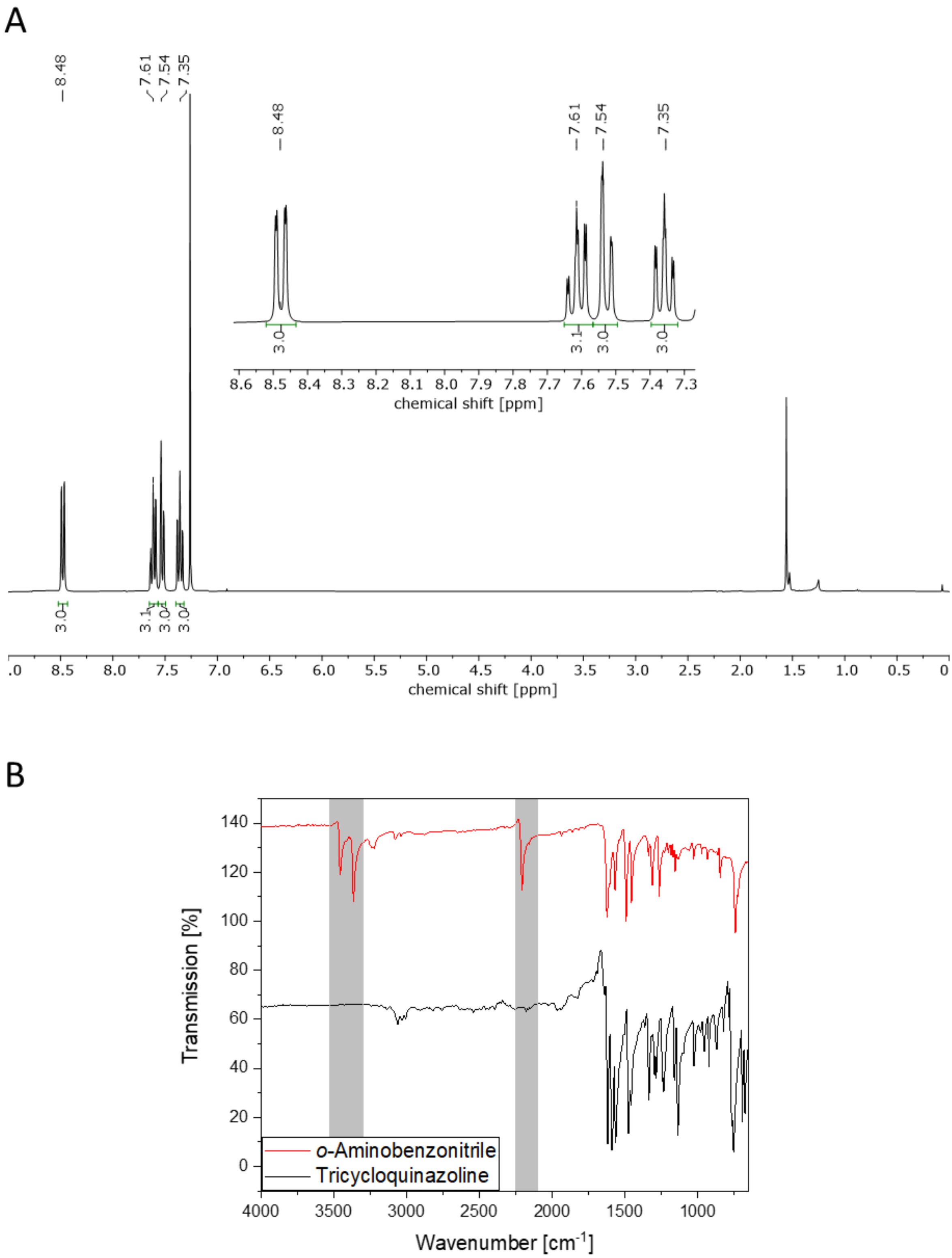

Figure S2. A: ^{1}H NMR of TCQ. The inset shows the aromatic region with the relevant protons. B: IR-Spectra of o-aminobenzonitrile (red) and TCQ (black). The grey areas indicate the regions of the nitrile (CN) stretching vibration at 2207 cm^{-1} and both amine (NH) stretching vibrations at 3366 cm^{-1} and 3458cm^{-1}, which can be clearly seen for the o-aminobenzonitrile and are not present in the TCQ spectra.

S3: Thermal characterization of TCQ

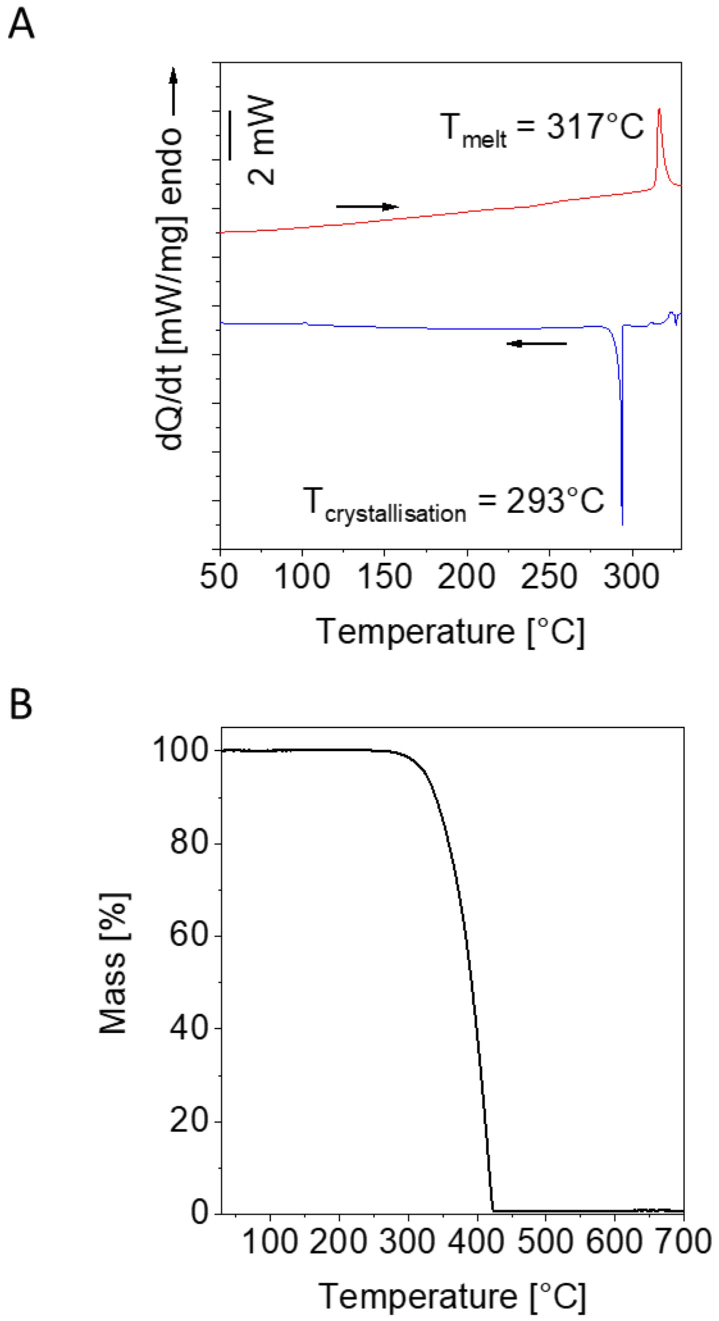

Figure S3. A: DSC measurements of TCQ from 30-330°C at a rate of 10 K min^{-1}; shown are the 2nd heating curve (red) and cooling curve (blue). B: TGA measurements of TCQ from 30-700°C at a heating rate of 10 K min^{-1} under N_2 with a flow rate of 30 mL min^{-1}.

S4: TEM micrograph and SAED of supramolecular TCQ nanofibers via PVD

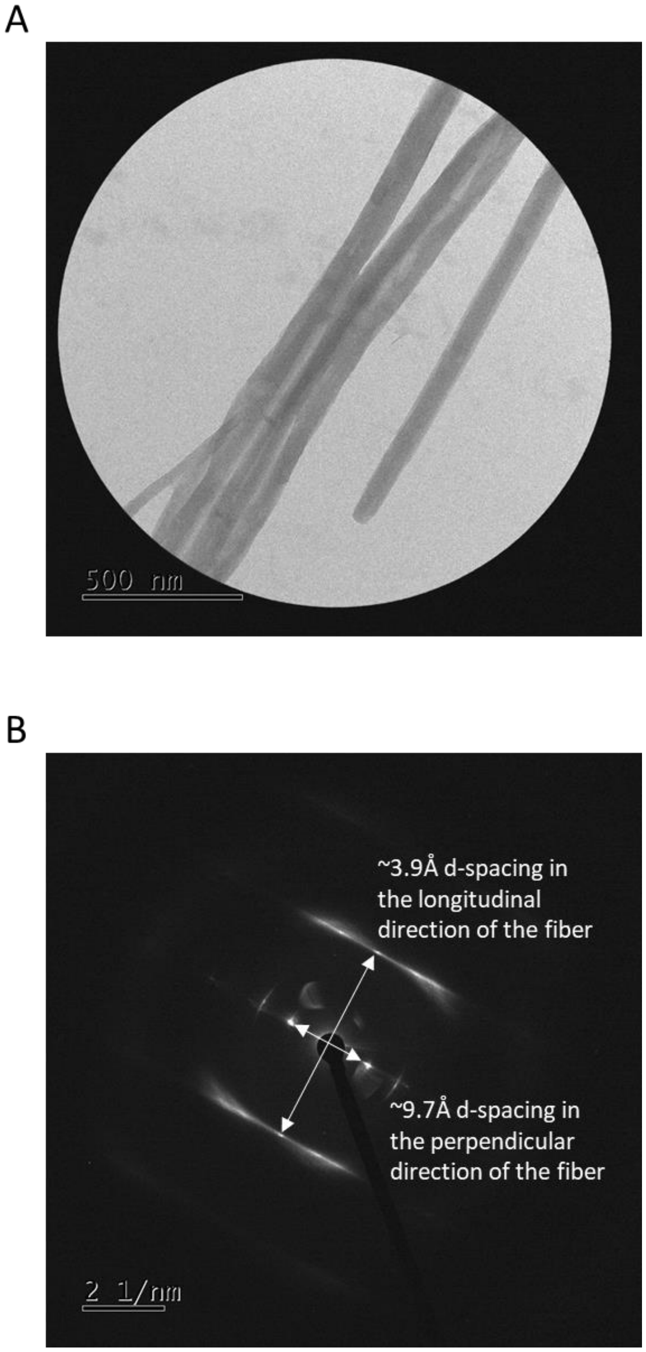

Figure S4. A: TEM image of TCQ nanofibers by PVD with a fiber diameter of 70 nm. Prior to TEM, TCQ nanofibers were scratched from a glass substrate and placed onto the TEM grid. B: Corresponding selected area electron diffraction (SAED) measurement of the nanofibers. In the SAED an inter disc distance of around 3.9 Å in the longitudinal direction of the fiber and a inter columnar distance of around 9.7 Å in perpendicular direction of the fiber can be observed.

S5: UV-vis characterization of TCQ in solution and TCQ nanofibers prepared via physical vapor deposition

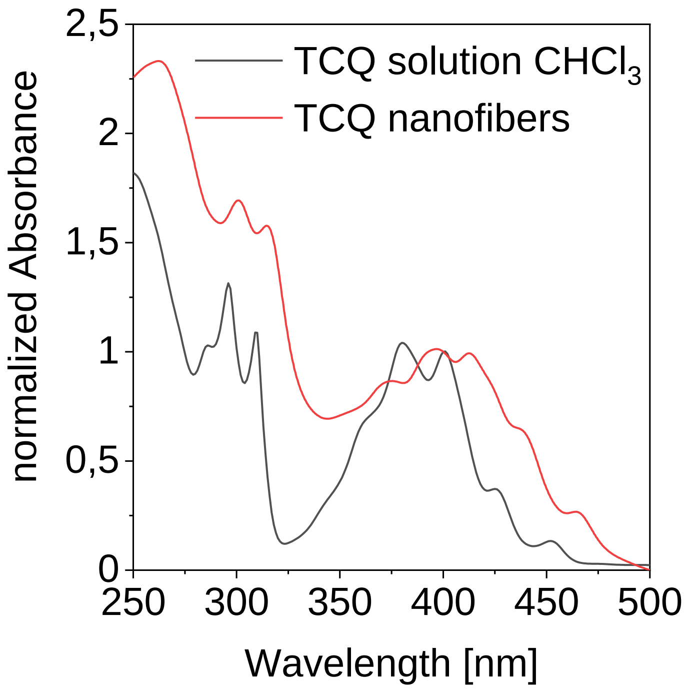

Figure S5. UV-vis absorption spectra of a 10^{-4} M TCQ solution in $CHCl_3$ (black) and a 1 µm thick supramolecular TCQ nanofiber mat on a quartz glass substrate prepared by PVD (red). For comparison, both spectra are normalized at 400 nm.

S6: Optical gap of supramolecular TCQ nanofibers

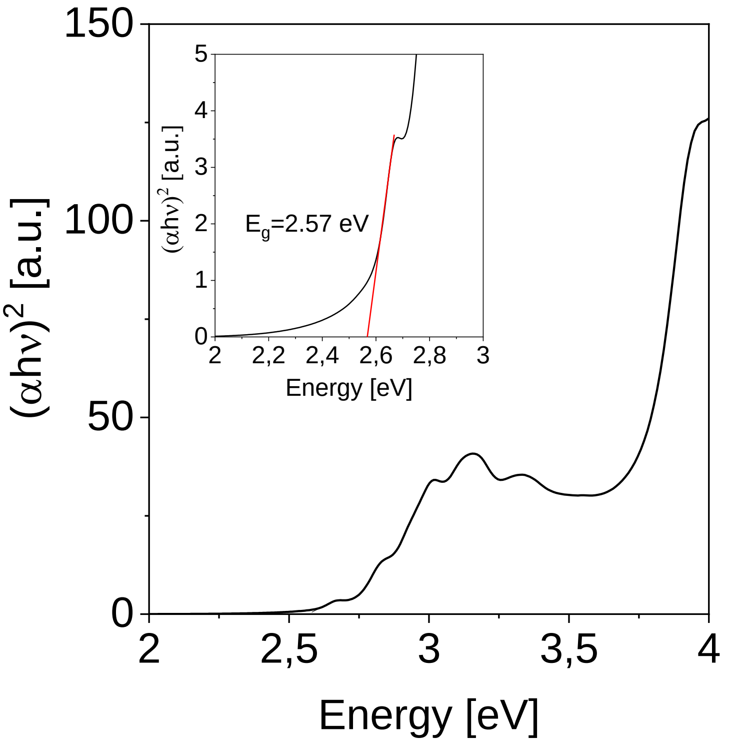

Figure S6. Tauc Plot of the supramolecular TCQ nanofibers physical vapor deposited on a quarz slide. The Inlet shows the onset of the optical gap which was determined to be 2.57 eV.

S7: Morphology investigations of physically vapor deposited TCQ nanofibers on different substrates

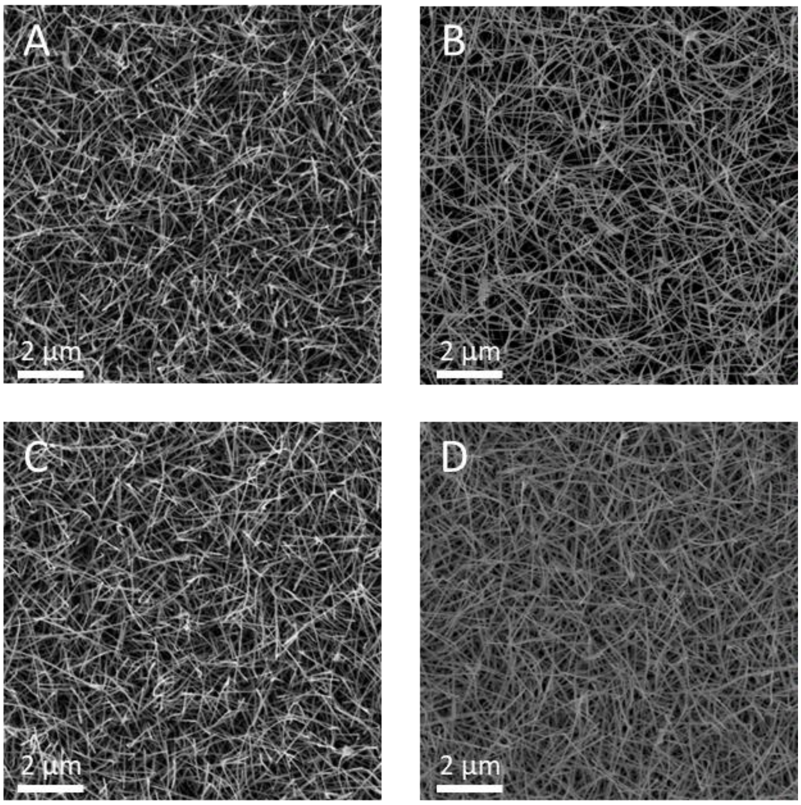

Figure S7. SEM images of TCQ supramolecular nanofibers prepared by PVD on different substrates. A) glass slide, B) quartz glass slide, C) plasma-etched glass slide and D) poly(lactic acid) substrate. For all substrates, a off-standing homogeneous fiber mat of TCQ nanofibers with a TCQ fiber diameter of 70 nm and a very similar densely packed morphology can be found. PVD conditions: T_{source} = 200°C, $T_{substrate}$ = 25°C, p = 10^{-6} mbar, deposition time = 300 s, evaporation rate = 3.3 nm/s.

S8: Photo- and micrograph characterization of the used glass microfiber nonwoven

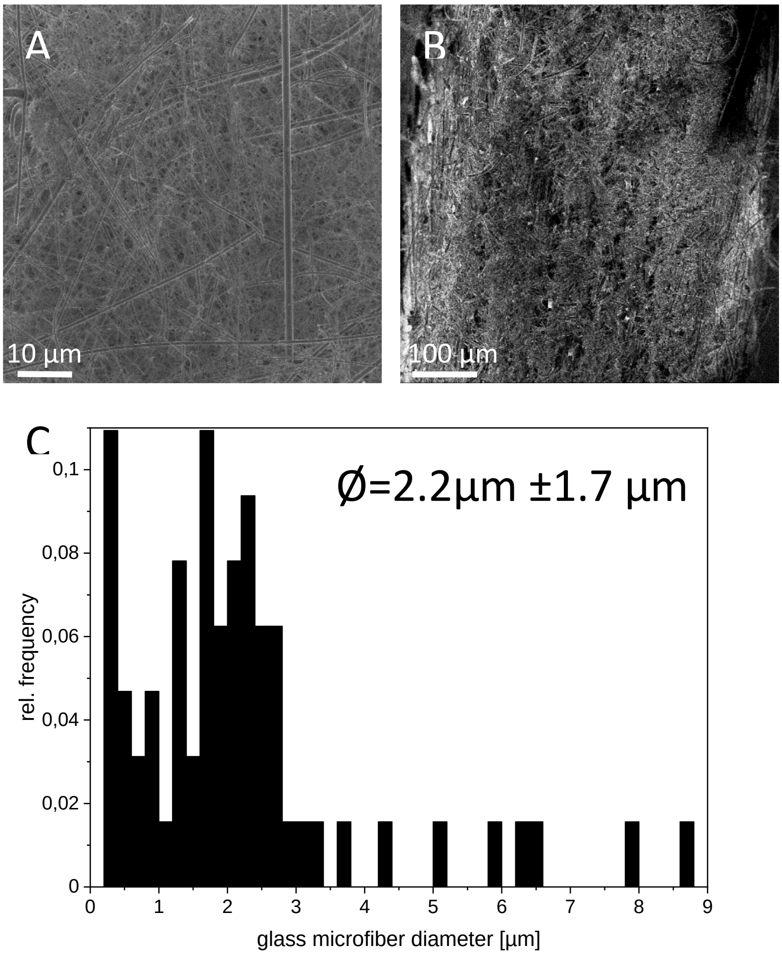

Figure S8. A and B: SEM images of a neat glass fiber nonwoven with a diameter of 37 mm and a thickness of 0.5 mm. C: Histogram of the glass microfiber diameters calculated by evaluating 100 fibers.

S9: Morphology investigations of supramolecular TCQ nanofiber/glass microfiber nonwovens

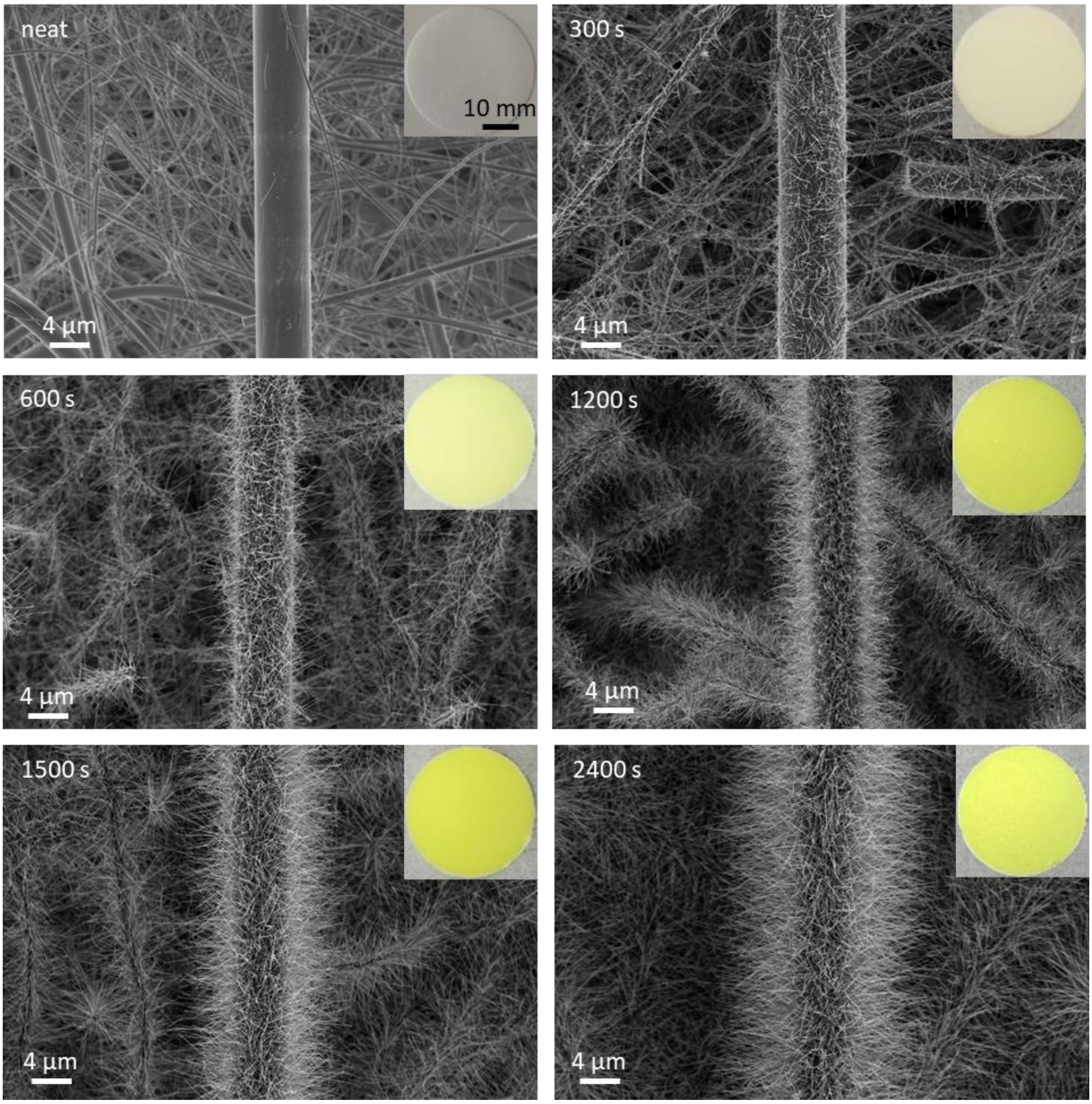

Figure S9. SEM images of neat glass fiber nonwoven and supramolecular TCQ nanofiber/ glass microfiber nonwovens by PVD with increasing deposition time from 300, 600, 1200, 1500 to 2400 seconds. The average diameter of the TCQ nanofibers for all mesostructured nonwovens were determined to be around 70 nm. The inlets show photographs of the mesostructured nonwovens with a macroscopic diameter of the specimen of 37 mm. PVD conditions: T_{source} = 200°C, $T_{substrate}$ = 25°C, p = 10^{-6} mbar, deposition time see figure, evaporation rate = 1.7 nm/s.

S10: HPLC Spectra before and after UV/light exposure tests of the supramolecular TCQ nanofiber/glass microfiber nonwoven

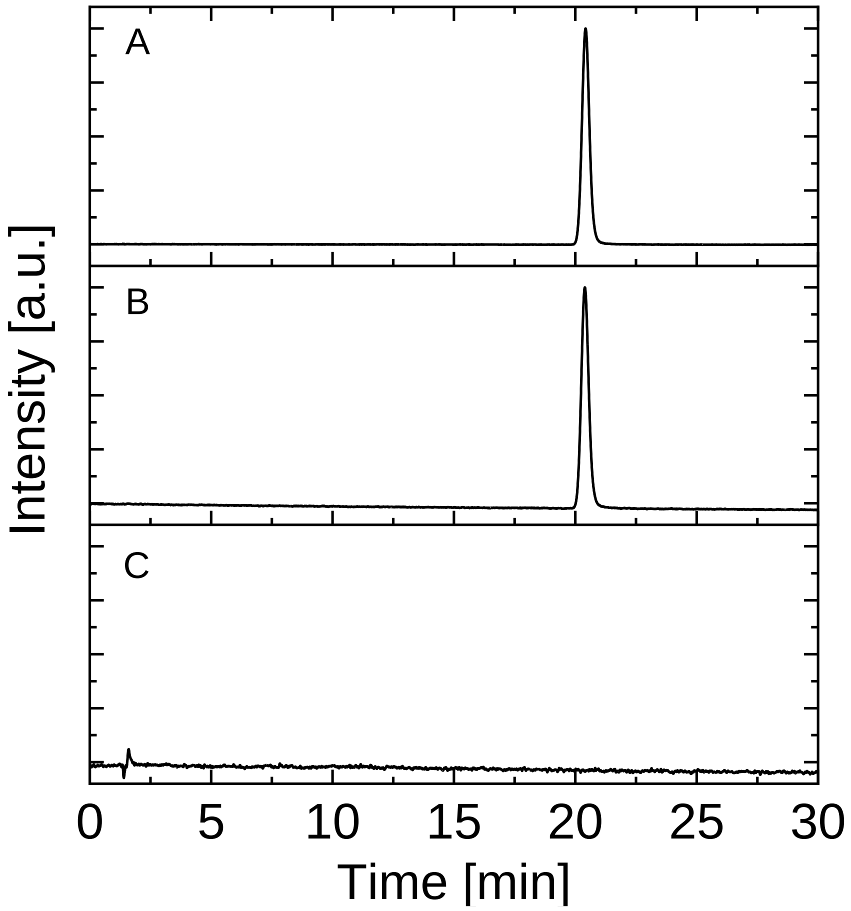

Figure S10. HPLC elugram of the supramolecular TCQ nanofiber/glass microfiber nonwoven immersed in water before (A) and after (B) UV/light exposure test. HPLC samples were prepared by immersing small pieces of the nanofiber/glass microfiber nonwoven in acetonitrile till the TCQ was completely dissolved. The water where the mesostructured nonwoven was immersed during the UV/light exposure test, was also analyzed (C) and did not show any TCQ in it. HPLC conditions: eluent: acetonitrile/water 70/30, flow rate: 1mL min^{-1}, injection volume: 10 µL.

S11: SEM images before and after UV/light exposure tests of the supramolecular TCQ nanofiber/glass microfiber nonwoven

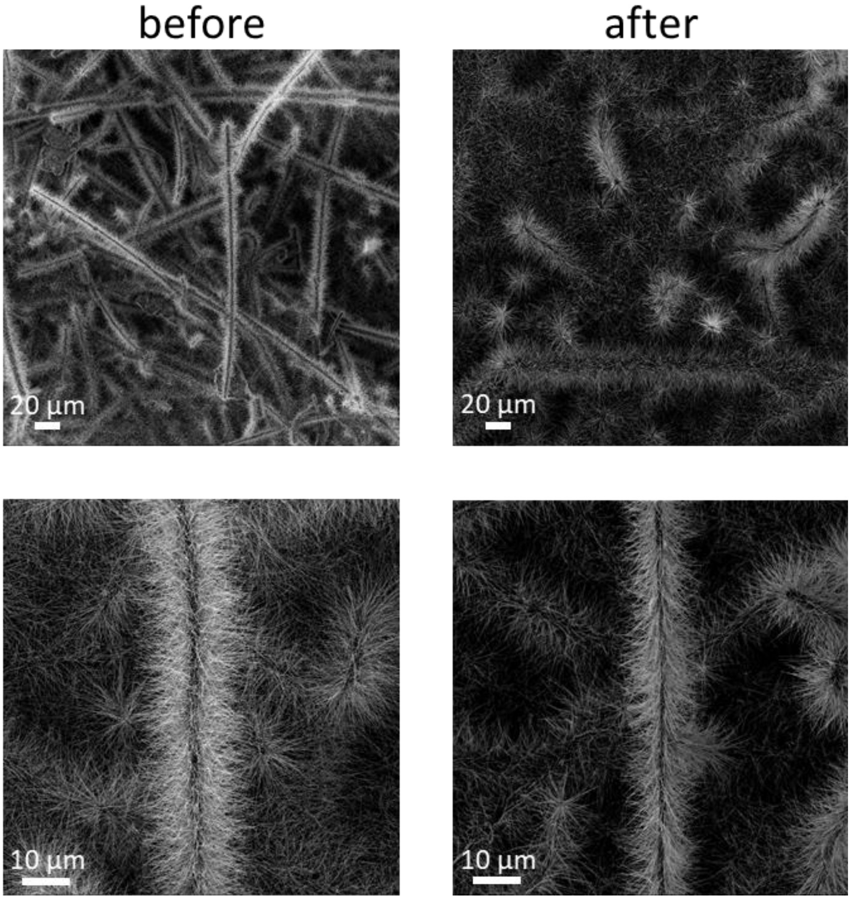

Figure S11. SEM images overview (top) and magnification (bottom) of supramolecular TCQ nanofiber/glass microfiber nonwovens before (left) and after (right) UV/light exposure tests. The illumination of the mesostructured nonwoven in water do not lead to a significant change of the morphology.

S12: Setups for photocatalytic degradation of Rhodamine B using mesostructured nonwoven

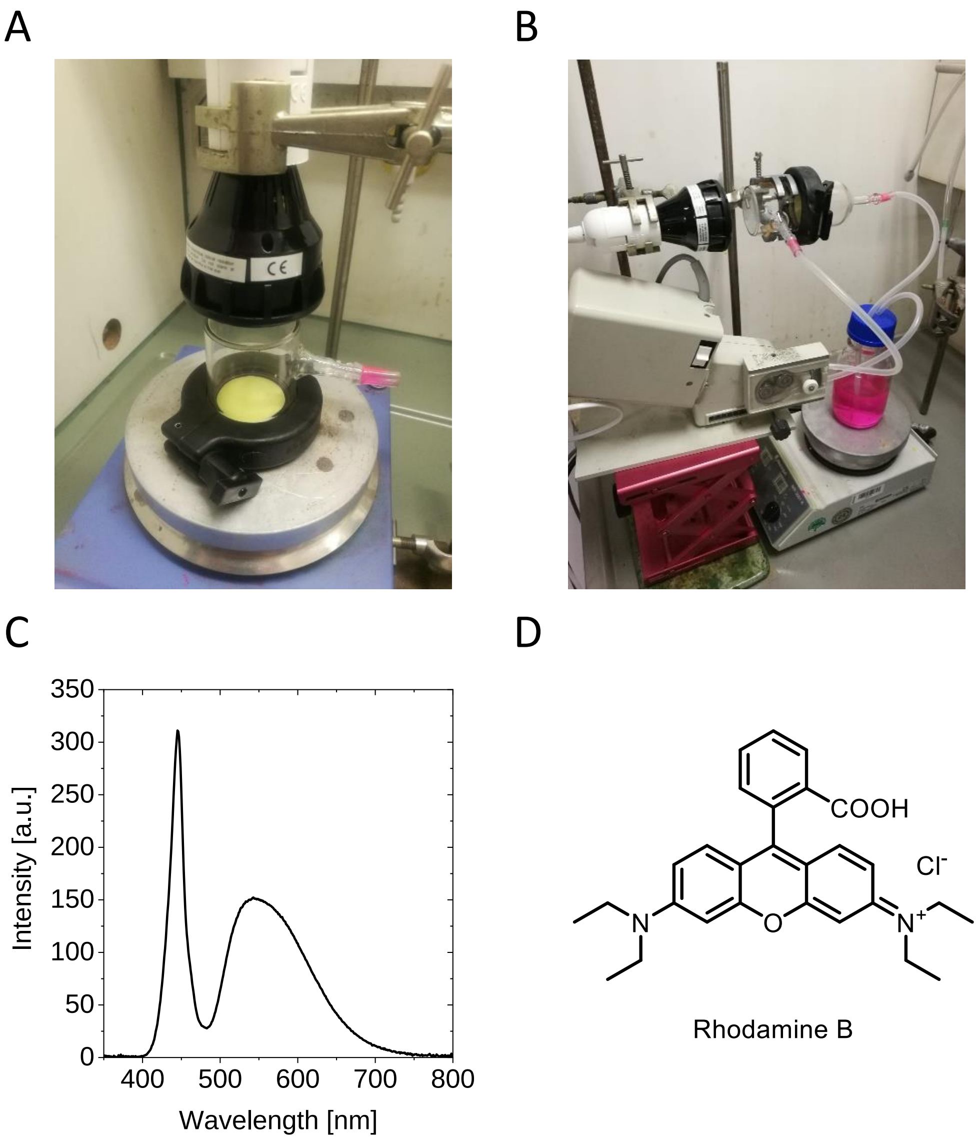

Figure S12. A: Batch setup for the degradation of Rhodamine B, B: Continuous flow setup for the degradation of Rhodamine B and Tetracycline. C: Emission spectra of the used LED light source. D: Molecular structure of Rhodamine B.

S13: Photocatalytic degradation of Rhodamine B using mesostructured nonwoven in a batch setup

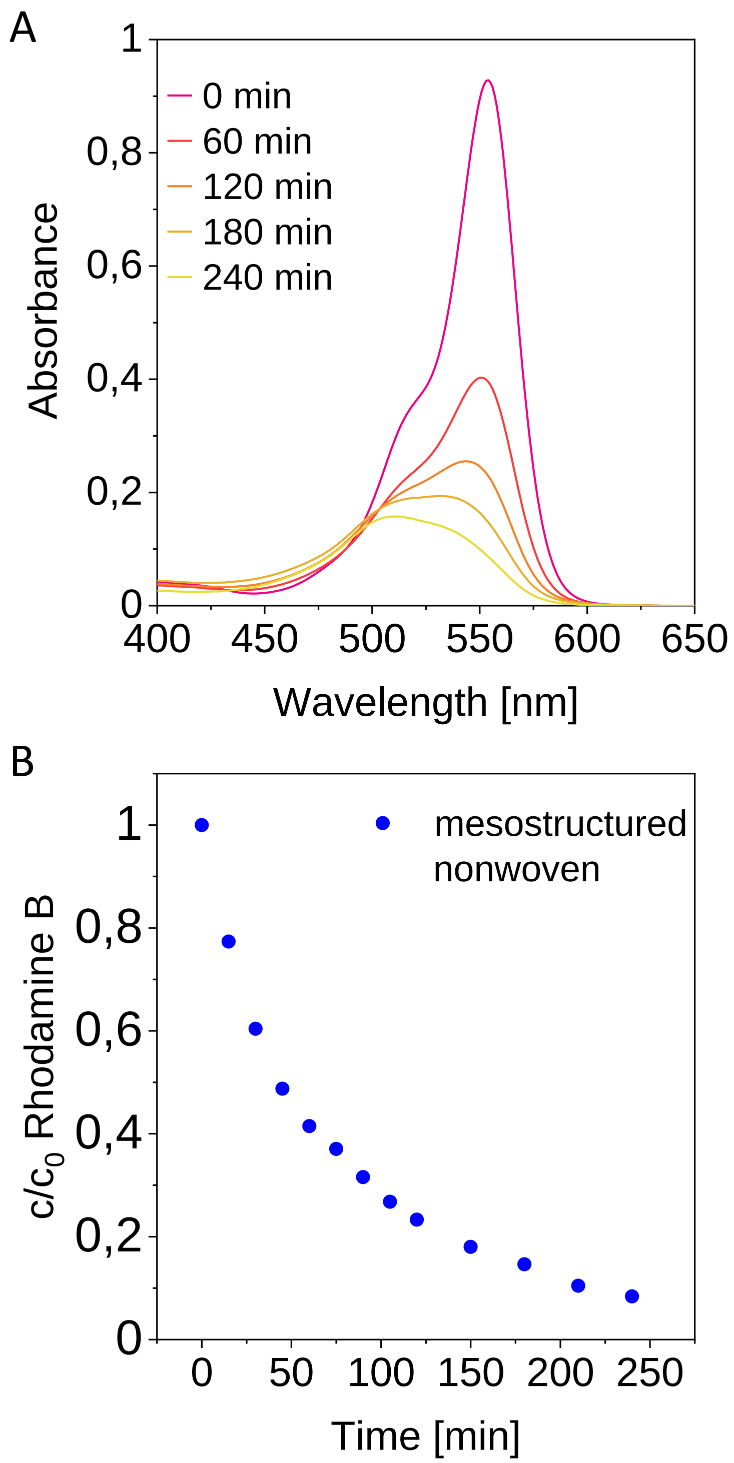

Figure S13. A: Progress of the UV-vis absorption of aqueous Rhodamine B solution during photocatalysis using a mesostructured nonwoven and a batch setup, B: Progress of photodegradation of Rhodamine B as a function of time using mesostructured nonwovens.

S14: Light on/off experiment during the photocatalytic degradation of Rhodamine B using mesostructured nonwoven in a batch setup

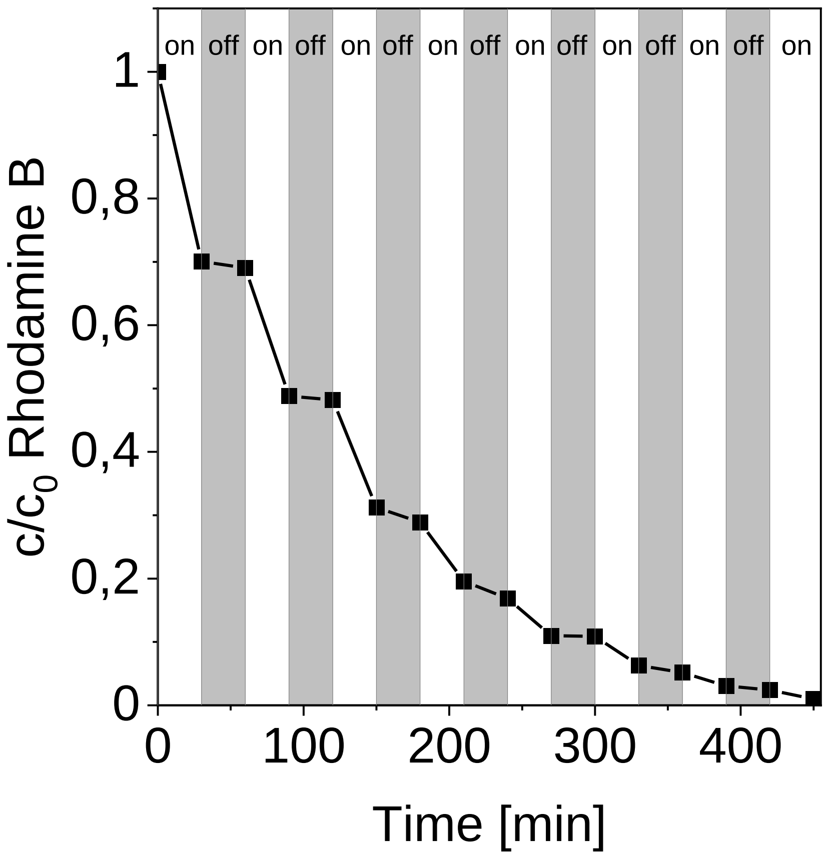

Figure S14. Progress of photodegradation of Rhodamine B as a function of time treating mesostructured nonwovens with successive 30 min periods of turning the light on and off for a total of 7.5 h.

S15: Morphology investigations of supramolecular nanofiber/glass microfiber nonwoven after photocatalysis in a batch setup

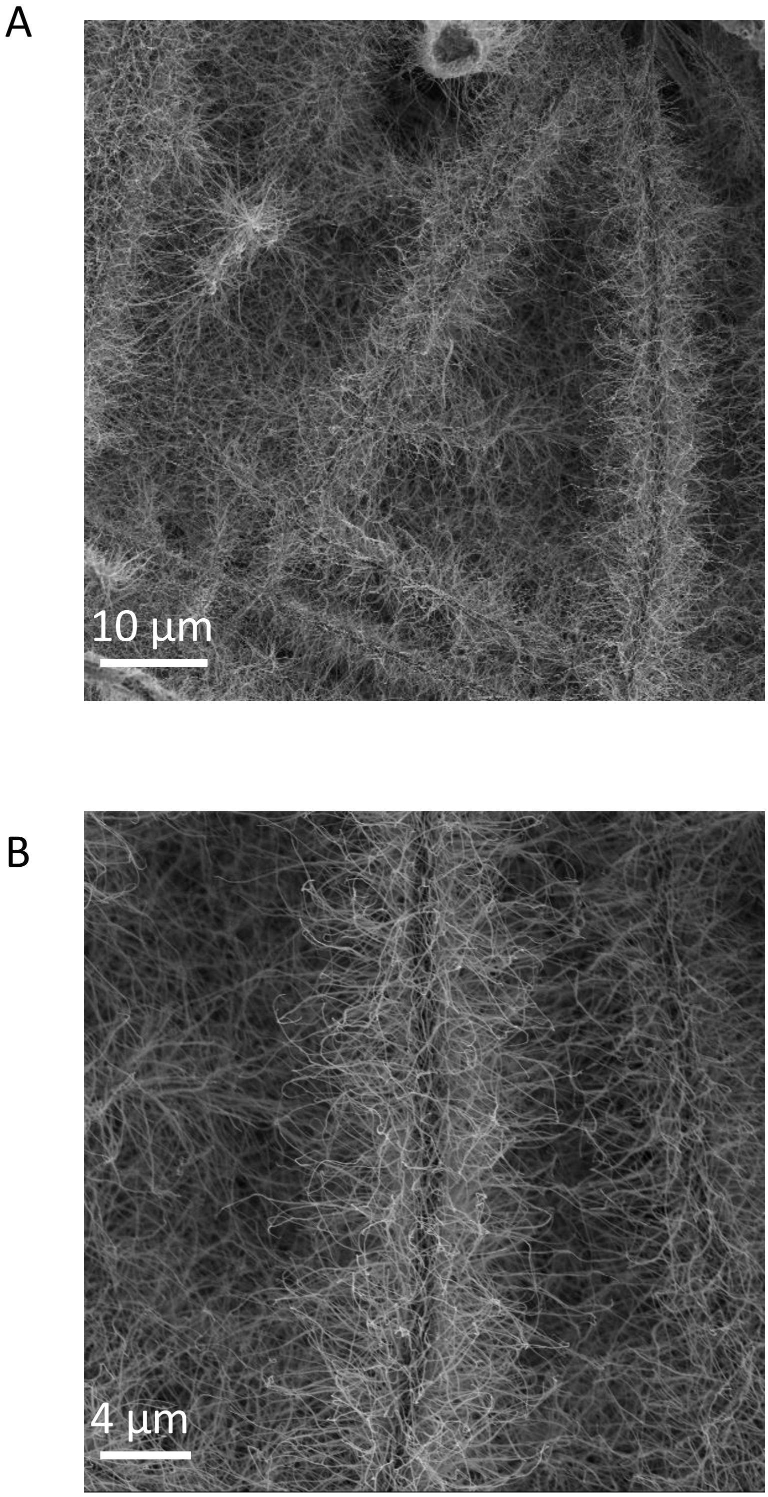

Figure S15. SEM image overview (A) and magnification (B) of TCQ nanofiber/glass microfiber nonwoven after 4 hours of photocatalysis in a batch reactor.

S16: Photocatalytic degradation of Rhodamine B using unsupported TCQ nanofibers in a batch setup

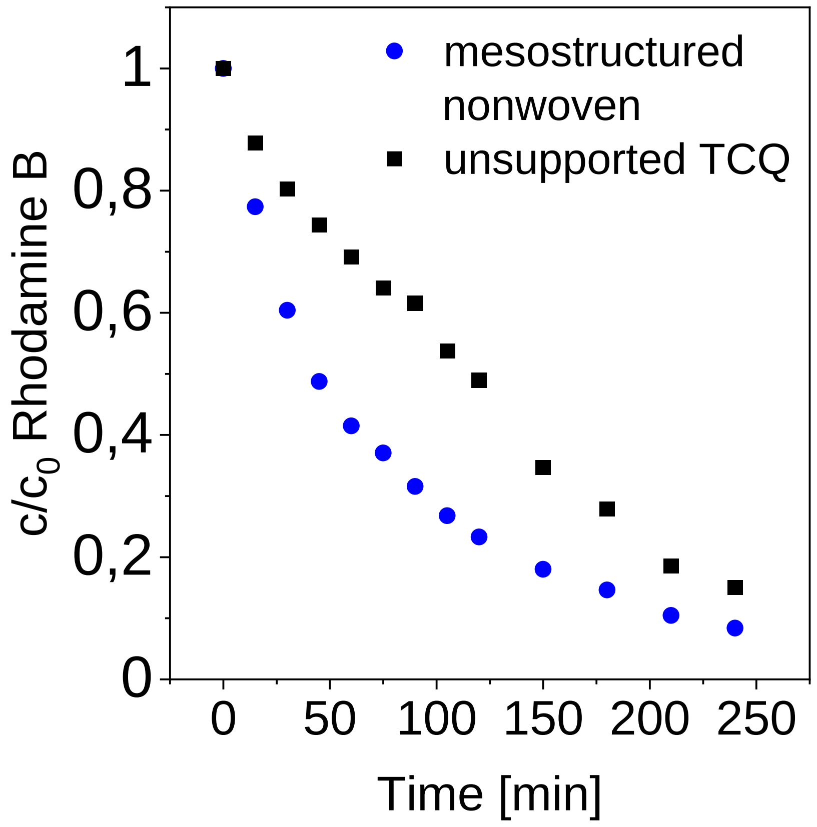

Figure S16. Progress of photodegradation of Rhodamine B as a function of time using unsupported TCQ nanofibers and a mesostructured nonwoven with the same amount of TCQ nanofibers for comparison.

S17: Reaction mechanism studies on the photocatalytic degradation of Rhodamine B using mesostructured nonwoven in a batch setup

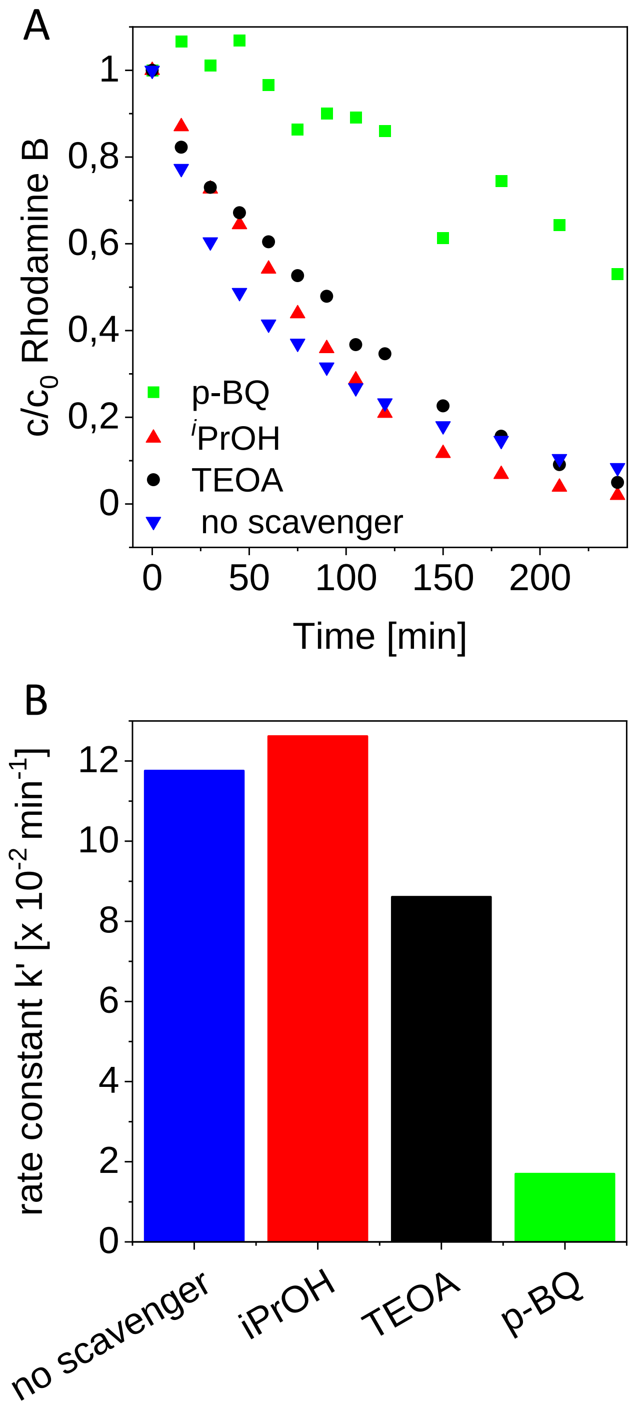

Figure S17. A: Progress of photodegradation of Rhodamine B as a function of time using mesostructured nonwovens in the presence of isopropanol (^{i}PrOH), triethanolamine (TEOA) and *p*-benzoquinone (*p*-BQ) that were used as radical scavengers for hydroxyl, hole and superoxide radicals, respectively. B: corresponding apparent rate constants k' assuming first order kinetics.

S18: Photocatalytic degradation of Rhodamine B using mesostructured nonwoven in a continuous flow setup

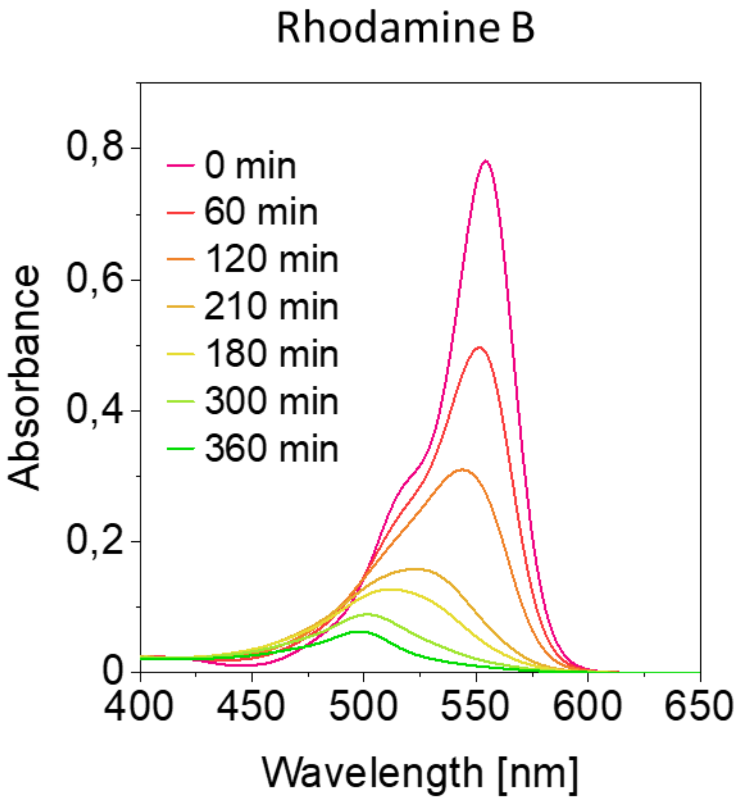

Figure S18. Progress of the UV-vis absorption of aqueous Rhodamine B solution during photocatalysis using a continuous flow setup.

S19: Leaching test of TCQ from the mesostructured nonwoven

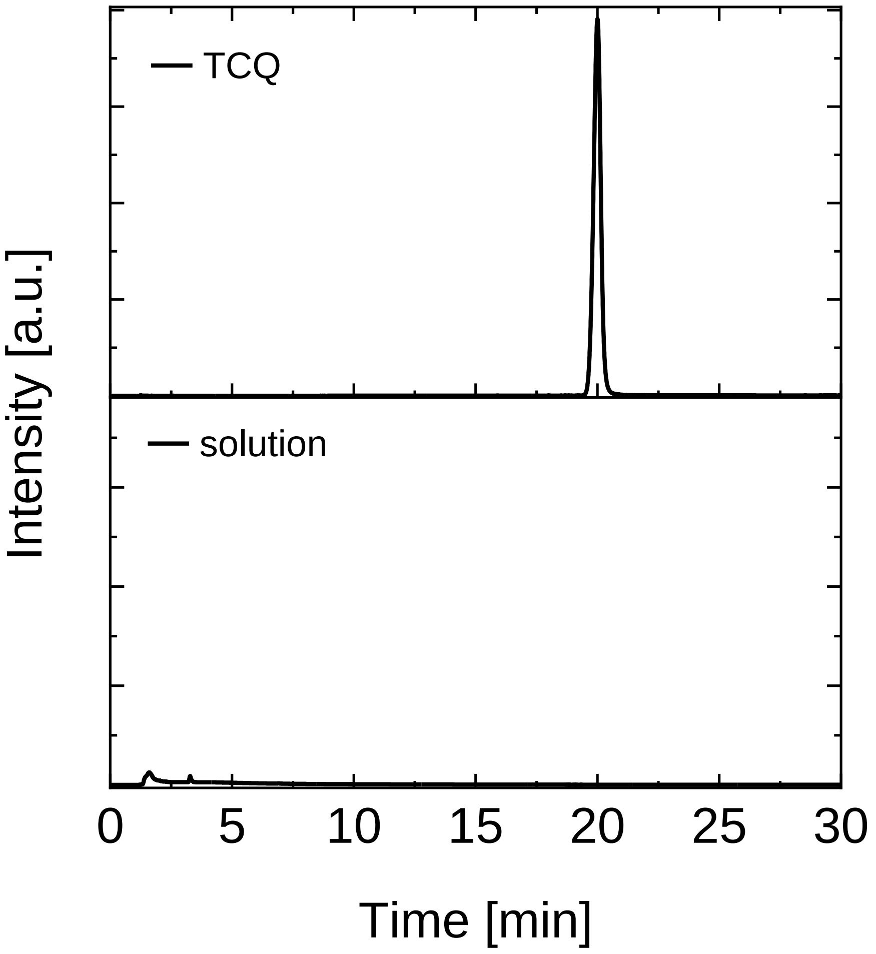

Figure S19. HPLC Chromatograms of TCQ (top) and the solution (bottom) after photodegradation of tetracycline that was circulated for 6h in the continuous-flow setup with a flow rate of 1.50 L/h, showing that no TCQ was leached or dissolved from the mesostructured nonwoven.

S20: Reusability test of supramolecular nanofiber/glass microfiber nonwoven

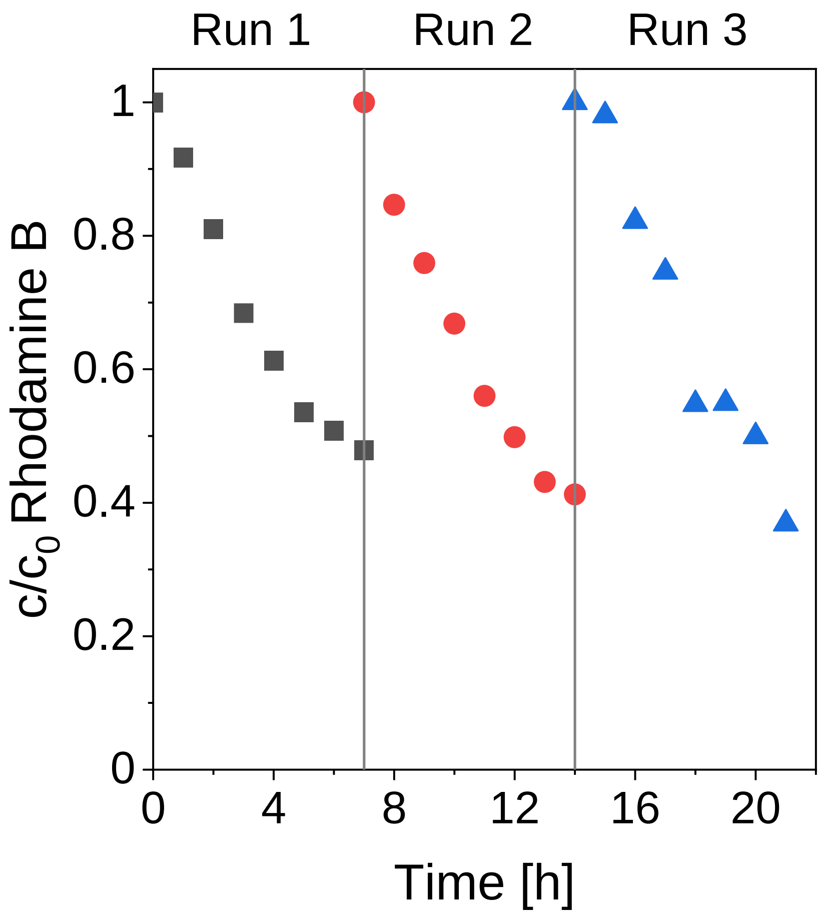

Figure S20. Reusability tests of TCQ mesostructured nonwoven in photocatalytic degradation of Rhodamine B. After each run, the mesostructured nonwoven was dried and reused in the continuous-flow setup, employing a fresh solution of Rhodamine B with the same concentration. Comparing the three runs, no significant loss in activity can be observed. Note that a mesostructured nonwoven with a TCQ loading of 0.015 mg cm^{-2} was used.

S21: Morphology investigations of supramolecular nanofiber/glass microfiber nonwoven after photocatalysis

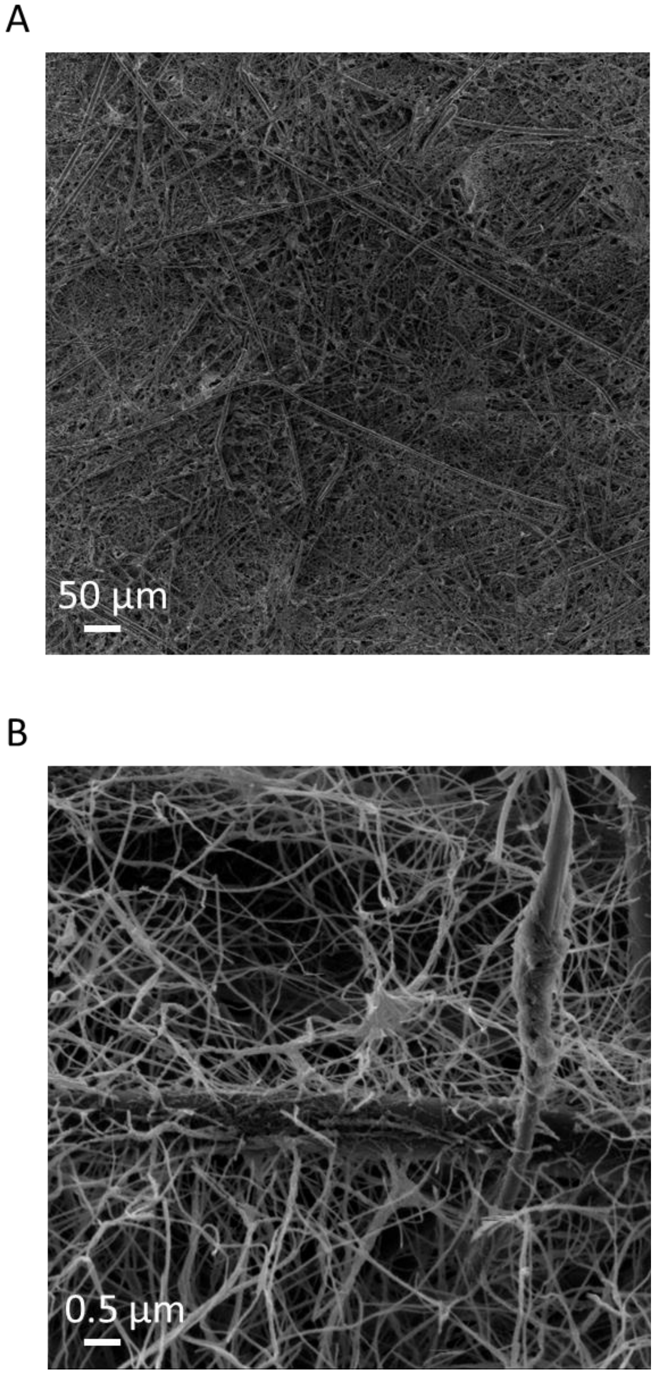

Figure S21. SEM image overview (A) and magnification (B) of TCQ nanofiber/glass microfiber nonwoven after three cycles (3x6 hours) of photocatalysis in a continuous-flow reactor with a flow rate of 1.50 L/h.

S22: HPLC investigations of supramolecular nanofiber/glass microfiber nonwoven after photocatalysis

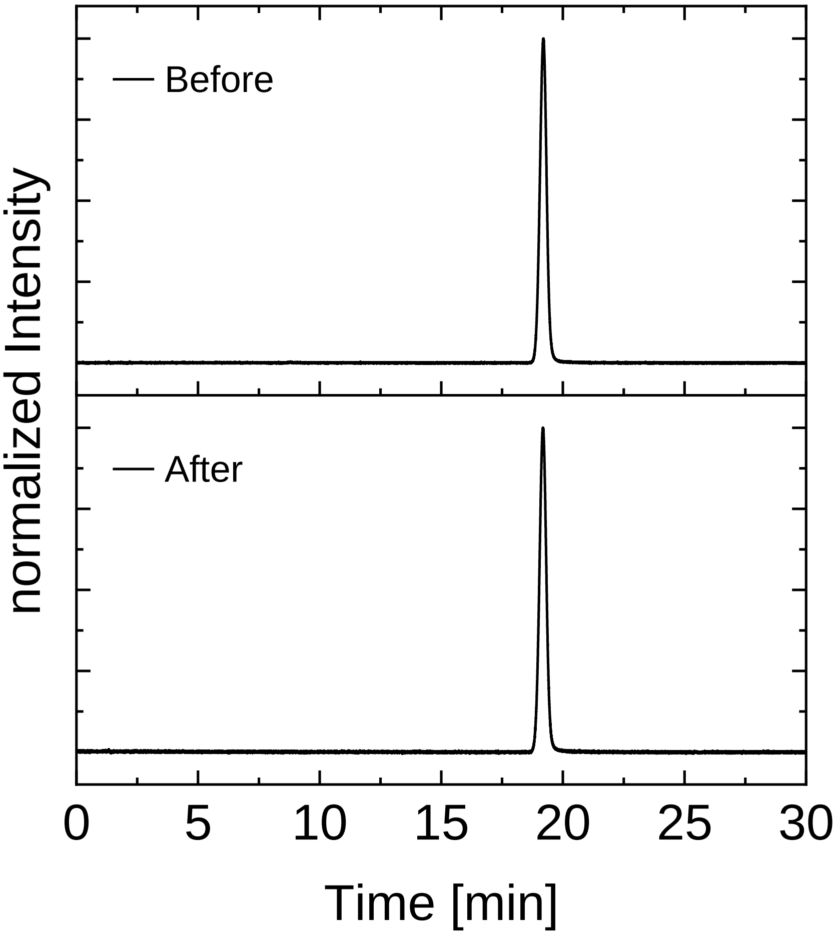

Figure S22. HPLC elugram of the supramolecular TCQ nanofiber/glass microfiber nonwoven before (top) and after (bottom) three runs of photocatalysis. HPLC samples were prepared by immersing small pieces of the nanofiber/glass microfiber nonwoven in acetonitrile till the TCQ was completely dissolved. HPLC conditions: eluent: acetonitrile/water 70/30, flow rate: 1mL min^{-1}, injection volume: 10 µL

S23: Photocatalytic degradation of Tetracycline using a continuous flow reactor

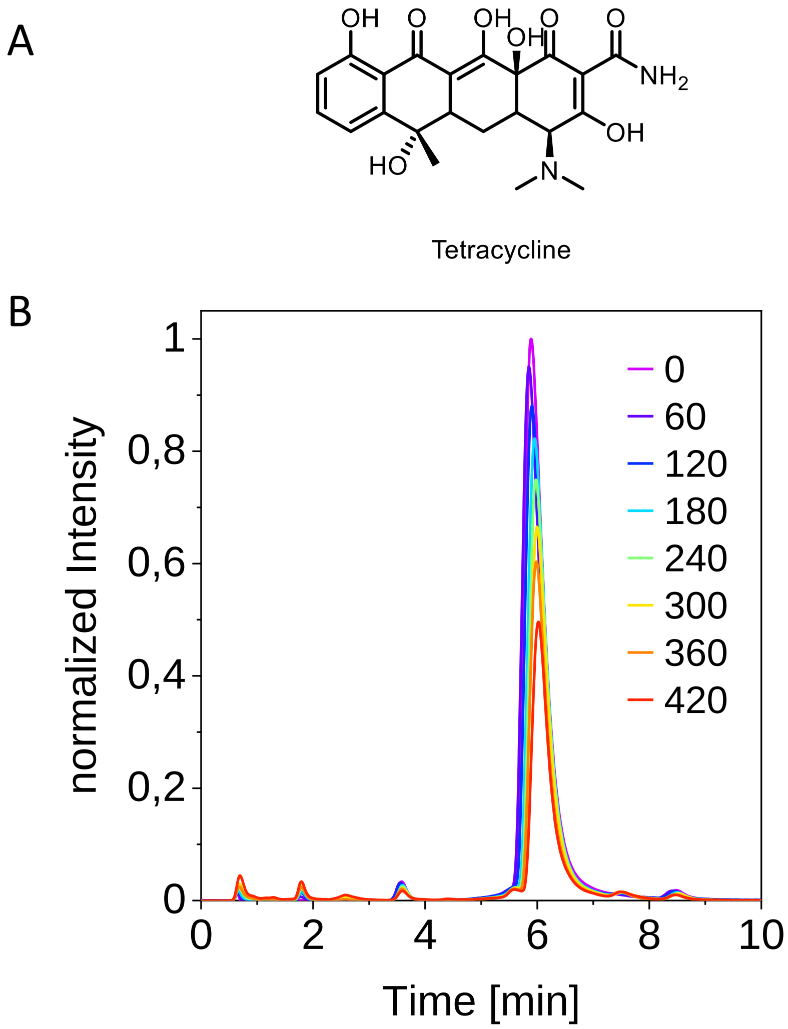

Figure S23.A: Molecular structure of tetracycline, B: HPLC elugram of aqueous solution of tetracycline during photocatalysis using a continuous flow setup at different irradiation times. HPLC conditions: eluent: water (1% formic acid)/MeOH 85/15, flow rate: 1mL min^{-1}, injection volume: 5 µL.
